# Functional Urate-Associated Genetic Variants Influence Expression of lincRNAs *LINC01229* and *MAFTRR*

**DOI:** 10.3389/fgene.2018.00733

**Published:** 2019-01-21

**Authors:** Megan Leask, Amy Dowdle, Hamish Salvesen, Ruth Topless, Tayaza Fadason, Wenhua Wei, William Schierding, Judith Marsman, Jisha Antony, Justin M. O’Sullivan, Tony R. Merriman, Julia A. Horsfield

**Affiliations:** ^1^Department of Pathology, Dunedin School of Medicine, University of Otago, Dunedin, New Zealand; ^2^Maurice Wilkins Centre for Molecular Biodiscovery, The University of Auckland, Auckland, New Zealand; ^3^Department of Biochemistry, School of Biomedical Sciences, University of Otago, Dunedin, New Zealand; ^4^Liggins Institute, The University of Auckland, Auckland, New Zealand; ^5^Department of Women’s and Children’s Health, Dunedin School of Medicine, University of Otago, Dunedin, New Zealand

**Keywords:** enhancer, eQTL, gout, HNF4A, lincRNA, *MAF*, non-coding, serum urate

## Abstract

Genetic variation in the genomic regulatory landscape likely plays a crucial role in the pathology of disease. Non-coding variants associated with disease can influence the expression of long intergenic non-coding RNAs (lincRNAs), which in turn function in the control of protein-coding gene expression. Here, we investigate the function of two independent serum urate-associated signals (SUA1 and SUA2) in close proximity to lincRNAs and an enhancer that reside ∼60 kb and ∼300 kb upstream of *MAF*, respectively. Variants within SUA1 are expression quantitative trait loci (eQTL) for *LINC01229* and *MAFTRR*, both co-expressed with *MAF*. We have also identified that variants within SUA1 are *trans*-eQTL for genes that are active in kidney- and serum urate-relevant pathways. Serum urate-associated variants *rs4077450* and *rs4077451* within SUA2 lie within an enhancer that recruits the transcription factor HNF4α and forms long range interactions with *LINC01229* and *MAFTRR*. The urate-raising alleles of *rs4077450* and *rs4077451* increase enhancer activity and associate with increased expression of *LINC01229*. We show that the SUA2 enhancer region drives expression in the zebrafish pronephros, recapitulating endogenous *MAF* expression. Depletion of *MAFTRR* and *LINC01229* in HEK293 cells in turn lead to increased *MAF* expression. Collectively, our results are consistent with serum urate variants mediating long-range transcriptional regulation of the lincRNAs *LINC01229* and *MAFTRR* and urate relevant genes (e.g., *SLC5A8* and *EHHADH*) in *trans*.

## Introduction

High levels of serum urate are necessary but not sufficient for gout, a debilitating form of inflammatory arthritis (Dalbeth et al., 2016). Hyperuricemia is a predictor of renal disease, cardiovascular disease and components of metabolic disease including obesity, fatty liver, diabetes, and hypertension (Sharaf El Din et al., 2017). Serum urate homeostasis is normally maintained by balancing urate production and excretion, predominantly by the liver and renal system, respectively (Maiuolo et al., 2016). Increased reabsorption of urate in the renal tubules (Hyndman et al., 2016) can cause high serum urate levels. Understanding the molecular mechanisms that control urate homeostasis is critical to improving the prevention, management, and treatment for individuals affected with gout and possibly other associated metabolic diseases.

Genome wide association studies (GWAS) have identified single nucleotide polymorphisms (SNPs) associated with serum urate levels (Tin et al., 2011; Okada et al., 2012; Köttgen et al., 2013; Kanai et al., 2018). However, the majority of these associated genetic variants lie outside of coding regions and the molecular mechanisms through which they alter urate levels are unknown. Nevertheless, association with serum urate levels indicates that these variants must be linked to or lie within regions of the genome that have biological consequences for serum urate control. To understand how genetic variants might influence disease traits it is important to: (1) identify the function of the DNA regions to which the disease-associated variants map; and (2) identify which gene(s) and pathways are under their control (if the function is regulatory). Previously, we used this approach to assign function to serum urate-associated variants within a conserved non-coding region that regulates expression of *PDZK1* (Ketharnathan et al., 2018).

**FIGURE 1:**
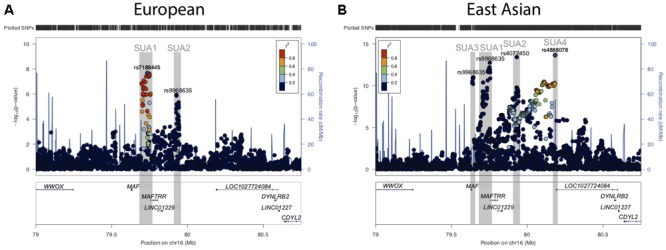
Serum urate associated regions upstream of the *MAF* gene. **(A)** Regional association plots for serum urate associated SNPs upstream of the *MAF* TSS and in proximity to the lincRNA region encompassing *MAFTRR, LINC01229*, and *LOC1027724084* from **(A)** Köttgen et al. (2013) and **(B)** Kanai et al. (2018). Surrounding genes include *WWOX, DYNLRB2* and *CDYL2*. Dots indicate individual SNPs while their position relative to the left *Y*-axis indicates the significance (-log_10_*P*) of association with serum urate levels. The blue line indicates the recombination rate across the locus. The SUA1, SUA2, SUA3, and SUA4 regions upstream of *MAF* are indicated by gray boxes and were defined by SNPs with *p* < 10^-6^ and LD with the lead SNP at the SUA region *R*^2^ > 0.8. The lead SNPs **(A)** *rs7188445* (SUA1) in Europeans and **(B)** *rs4888078* (SUA4) in East Asians are indicated by a purple dot. The color of the surrounding SNPs indicates the strength of LD with the lead SNP according to the key in the left top hand corner, measured as *r^2^* found in the HapMap data (hg19/1000 genomes Nov 2014) for Europeans **(A)** and East Asian **(B)**. The lead SNP of each SUA region is indicated above the signal in Europeans **(A)** and East Asians **(B)**. The plot was generated using LocusZoom (Pruim et al., 2010).

The *MAF* bZIP transcription factor (*MAF*) is a strong candidate gene for involvement in serum urate control and gout. *MAF* is highly expressed in the human and mouse kidney, including the podocytes (Daassi et al., 2016) and proximal tubules (Imaki et al., 2004; Ponten et al., 2008). MAF function is important for the development and differentiation of the proximal tubule cells (Imaki et al., 2004). Additionally MAF has been shown to regulate antioxidant (Dhakshinamoorthy and Jaiswal, 2002; Kikuchi et al., 2018) and apoptotic pathways (Peng et al., 2007) that are required for NLRP3 inflammasome activation implicated in chronic kidney disease and gout (Jhang and Yen, 2017; So and Martinon, 2017). Although there are multiple genetically independent signals associated with serum urate levels in a ∼500 kb region upstream of the *MAF* gene (Figure 1), it is not known if these influence the expression of *MAF*.

The urate association signals that are located upstream of *MAF* are in close proximity to multiple long intergenic non-coding (linc) RNAs. Recent evidence has shown that non-coding disease-associated SNPs can regulate the expression of lincRNAs (Kumar et al., 2013; Mirza et al., 2014). As a direct result of changing lincRNA expression, disease-associated SNPs could have indirect tissue-specific effects on protein-coding genes (Tan et al., 2017) that are involved in disease. Here, we investigate how serum urate-associated variants in two prominent urate-association signals (SUA1 and SUA2) that are conserved between GWAS in European and Japanese sample sets could contribute to the regulation of serum urate levels.

## Results

In people of European ancestry, two loci located upstream of *MAF* [Chr16: 79637239–79645062 (build 37.7)] were identified as being associated with serum urate levels *(p* < 10^-6^, hereafter referred to as serum urate association regions 1 (SUA1) and 2 (SUA2) (Figure 1A and Table 1) (Köttgen et al., 2013). SUA1 is located ∼60 kb upstream of the *MAF* transcriptional start site (TSS) and extends through to the 3′ region of *MAF transcriptional regulator RNA* (*MAFTRR*). The lead SUA1 SNP (*rs7188445*) is associated with gout (Phipps-Green et al., 2016). SUA2 is ∼300 kb upstream of the *MAF* locus and ∼120 kb upstream of the TSS of *long intergenic non-protein coding RNA 1229* (*LINC01229*) (Figure 1A). SUA1 and SUA2 have also been associated with serum urate levels in East Asian datasets (Okada et al., 2012; Kanai et al., 2018) [Table 1 and Figure 1B (data from Kanai et al., 2018)]. A third serum urate association signal (SUA3) (Table 1 and Figure 1B) located immediately upstream of *MAF* has currently only been identified in East Asian serum urate datasets (Okada et al., 2012; Kanai et al., 2018). The lead Okada et al. (2012) SUA3 SNP (*rs889472*) is associated with gout (Higashino et al., 2018) and is in nearly perfect linkage disequilibrium (LD) (*R*^2^ = 0.97) with the lead SUA3 SNP (*rs8050348*) in the Kanai et al. (2018) dataset (Figure 1B). A fourth serum urate association signal (SUA4) (Table 1 and Figure 1B) is only evident in the Kanai et al. (2018) Japanese data and is located immediately upstream of *LOC1027724084* (Kanai et al., 2018). The maximally associated variants (*p* = 2.3 × 10^-14^) at SUA4 in the Kanai et al. (2018) data (*rs4888080, rs4888078*, and *rs12325508*) are in LD (*R*^2^ = 1). The urate-raising alleles for these SNPs have allele frequencies of 0.03 in East Asian individuals from the 1000 Genomes Project and these SNPs are monomorphic in Europeans which explains the absence of the SUA4 signal from the European serum urate datasets. The region upstream of *MAF* has also been associated with other phenotypic traits (Table 1 and Supplementary Figure S1A). It is unknown whether variants in SUA1-4 affect the expression of *MAF*, or regulate other genes, including the lincRNAs at this region. In this study, we focussed on assigning function to the two independent regions SUA1 and SUA2 (determined by conditional analysis, Supplementary Figure S1B) that have been identified within both European and East Asian populations and are therefore conserved between population groups. SUA3 and SUA4 were excluded from our study due to their absence in the European population.

**Table 1:** Serum urate associated regions upstream of *MAF.*

	SUA1	SUA2	SUA3	SUA4
Position (Chr16; Hg19)	79696939–79756197	79924857–79945421	79637239–79645062	80183736–80184313
Region length	∼60 kb	∼20 kb	∼8 kb	578 bp
Distance to *MAF* TSS	-60 kb	-290 kb	-9 Kb	-550 Kb
**Lead urate increasing allele (frequency)**				
Köttgen et al., 2013	*rs7188445_G* (EUR = 0.65, EAS = 0.71)	*rs9935686_A* (EUR = 0.13, EAS = 0.16)	N/A	N/A
Kanai et al., 2018	*rs144899207_T* (other = 0.70, EAS = 0.70)	*rs4077450_G* (EUR = 0.16, EAS = 0.39)	*rs8050348_T* (EUR = 0.38, EAS = 0.62)	*rs4888078_*C (EUR = 0.00, EAS = 0.03)
Other associated phenotypes (*p* < 10^-4^)	Gout (Phipps-Green et al., 2016), thyroid related traits (Teumer et al., 2011; Porcu et al., 2013; Zhan et al., 2014), alkaline phosphatase levels, electrolyte phosphorous levels (Kanai et al., 2018)	Hemoglobin, hematocrit, serum creatinine and estimate glomerular filtration rate (Kanai et al., 2018)	BMI (male) (Kanai et al., 2018), gout (Higashino et al., 2018)	Gamma-glutamyl transferase (Kanai et al., 2018)

### SUA1 and SUA2 Colocalize With eQTL for *MAFTRR* and *LINC01229*

**FIGURE 2:**
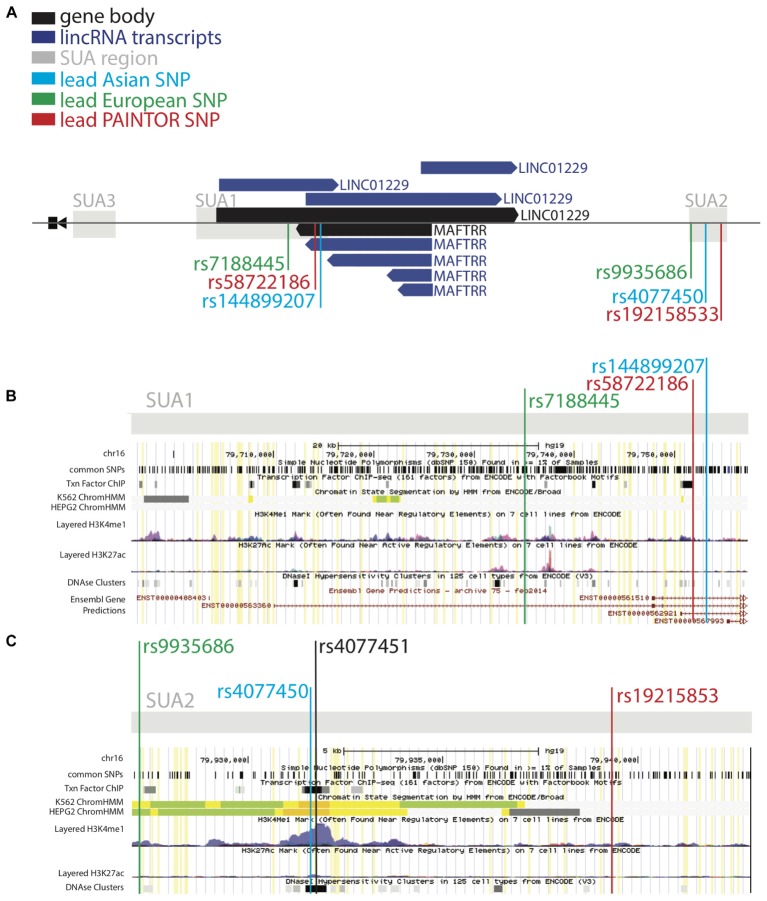
SUA2 has hallmarks of enhancer function. **(A)** The *MAF* upstream region marked by SUA variants in Köttgen et al. (2013) and Kanai et al. (2018). LincRNA gene bodies (black) and transcripts (dark blue) are depicted. **(B,C)** ENCODE screenshots of the SUA1 and SUA2 regions. The presence of enhancer elements; DNaseI hypersensitivity regions (DHS) (Thurman et al., 2012), H3K27ac and H3K4me1 chromatin modifications (Heintzman et al., 2009), transcription factor binding sites (Wang et al., 2012) and predicted ChromHMM enhancers (Ernst and Kellis, 2017) are shown. Yellow lines indicate Köttgen SUA variants (*p* < 10^-5^) **(B)** A schematic of the ENCODE annotation of the SUA1 region, which includes the lead SUA1 PAINTOR SNP *rs58722186* (red) lead European urate SNP *rs7188445* (green) and lead Japanese urate SNP *rs144899207* (blue). **(C)** A schematic of the ENCODE annotation of the SUA2 region, which includes the lead SUA2 PAINTOR SNP *rs192158533* (red) lead European urate SNP *rs9935686* (green) and lead Japanese urate SNP *rs4077450* (blue).

We used COLOC (Giambartolomei et al., 2014) and expression data from The Genotype Tissue Expression (GTEx) project (Carithers and Moore, 2015) to identify *cis*-eQTL that colocalize with SUA1 and SUA2. eQTL for the genes *MAFTRR* and *LINC01229* (Figure 2, gene body depicted in black) colocalize with the SUA1 urate signal (Table 2) although the lead eQTL variants are not the lead urate variants at SUA1 (Supplementary Figure S2). SUA1 urate-raising alleles associate with increased expression of *MAFTRR* but with lowered expression of *LINC01229* (e.g., *rs7188445_G* Supplementary Figure S3 and Supplementary Table S1). An eQTL for *LINC01229* in the GTEx tissue Skin – Exposed (Lower leg) (Supplementary Figure S2) colocalises with the SUA2 urate signal (Table 2). Urate-raising variants within SUA2 increase *LINC01229* expression (Supplementary Table S2). Colocalization of the SUA1 and SUA2 urate signals with *MAFTRR* and *LINC01229* eQTL suggests that the causal variant(s) at these regions for serum urate and lincRNA expression are shared. Collectively these results are consistent with expression of *MAFTRR* and *LINC01229* being functionally important in serum urate control.

**Table 2:** Colocalized GTEx eQTL and SUA upstream of *MAF.*

SUA region	eGene	PPC^1^	GTEx eQTL tissue	Effect of urate-raising alleles	GTEx single tissue eGene *p*-value
SUA1	*MAFTRR*	0.92	Pancreas	+	1.34 × 10^-8^
SUA1	*MAFTRR*	0.84	Colon (sigmoid)	+	3.00 × 10^-12^
SUA1	*LINC01229*	0.92	Testis	-	8.53 × 10^-3^
SUA2	*LINC01229*	0.76	Skin exposed (lower leg)	+	1.46 × 10^-97^

*^*1*^Posterior probability of colocalization (PPC) as determined by COLOC.*

*MAFTRR* eQTL variants (all GTEx tissues) that have the greatest effect on *MAFTRR* expression lie immediately 3′, or within the 3′ region of the *MAFTRR* transcript (Supplementary Figure S2). By contrast, the locations of eQTL for *LINC01229* differ in genomic location between tissues (Supplementary Figure S2). eQTL for *MAF* are not found within SUA1 or SUA2 and instead lie within the *WWOX* transcript (+375 kb of *MAF*), downstream and within the *MAF* transcript, and within the promoter of *DYNLRB2* (-1 Mb of *MAF*) (Supplementary Figure S4).

GTEx currently has no renal eQTL data, therefore we queried the NepheQTL database (Gillies et al., 2018) to assess whether the SUA1 and SUA2 *MAFTRR* and *LINC01229* eQTL exist in the kidney tubule. *MAFTRR* has a very strong eQTL signal (FDR adjusted *p* = 8.12 × 10^-18^) in the tubulointerstitium (Supplementary Figure S2) that coincides with SUA1. Four maximally associated variants (*R*^2^> 0.98) lie immediately 3′ to the *MAFTRR* transcript consistent with the GTEx data. COLOC indicates that the eQTL signal for *MAFTRR* and the SUA1 serum urate signal are different (H3 posterior probability = 0.976). Nonetheless, SUA1 urate-raising variants associate with increased expression of *MAFTRR* in kidney tubule (Supplementary Table S1). No SUA2 variants were eQTL for *MAFTRR* in the kidney tubules within the NepheQTL database (Gillies et al., 2018) (Supplementary Table S2). Furthermore, there were no significant gene eQTL signals for *MAF* or *LINC01229* within NepheQTL.

An additional whole kidney-derived eQTL dataset (Ko et al., 2017) and Japanese blood eQTL sample set^1^ [monocytes, CD4+, CD8+, natural killer and B cells (Kanai et al., 2018)] were queried. eQTL for *MAF* were located downstream of *MAF* (consistent with the GTEx data), however, there were no eQTL data for *MAFTRR* and *LINC01229* and no SUA (SUA1-4) variants were significant eQTL in these tissues (Supplementary Figure S5).

### SUA1 Serum Urate Variants Are Unlikely to Regulate Transcription

Intergenic disease-associated variants could influence disease traits by regulating gene expression. We used PAINTOR (Kichaev et al., 2014) in conjunction with ENCODE (Encyclopedia of DNA Elements) (ENCODE Project Consortium, 2012) to identify functional variants with regulatory activity at the SUA regions upstream of *MAF.* Fine mapping with PAINTOR using kidney cell type annotations (Finucane et al., 2015) as a prior identified the most likely causal variants within SUA1 and SUA2 (Table 3). We interrogated ENCODE to identify putative regulatory elements that overlap the SNPs identified by PAINTOR (Figure 2, Supplementary Tables S1, S2, and Supplementary Figures S6, S7). Functional annotations of the human genome were scanned to identify DNaseI hypersensitivity regions [DHS; characteristic of enhanced chromatin accessibility (Thurman et al., 2012)], the coexistence of histone H3 monomethylated on lysine 4 and histone H3 acetylated on lysine 27 (H3K4me1, H3K27ac; hallmarks of active *cis*-regulatory elements) (Heintzman et al., 2009) and transcription factor binding sites (Wang et al., 2012, 2013). We also looked for hyper-accelerated regions (HARs), which represent conserved genomic loci with elevated divergence in humans (Doan et al., 2016) and can therefore indicate potential human-specific enhancers.

**Table 3:** PAINTOR analysis of SUA1 and SUA2.

Variant ID	SUA region	PAINTOR posterior probability	Köttgen *p*-value	Kanai *p*-value	EUR MAF	EAS MAF
*rs192158533*	SUA2	0.89	4.6 × 10^-6^	N/A	0.01	0.00
*rs58722186*	SUA1	0.31	4.3 × 10^-8^	7.10 × 10^-13^	0.34	0.27
*rs4077450*	SUA2	0.27	8.29 × 10^-6^	3.9 × 10^-14^	0.16	0.39
*rs7188445*	Lead SUA1 SNP	0.064	2.08 × 10^-8^	3.4 × 10^-9^	0.35	0.29
*rs9935686*	Lead SUA2 SNP	0.059	1.14 × 10^-6^	1.0 × 10^-5^	0.13	0.16

Our ENCODE analyses indicate that *rs58722186* within SUA1 identified by PAINTOR (Table 3, Figures 2A,B, PAINTOR posterior probability = 0.31) is in close proximity to regulatory elements. *rs58722186* is adjacent to five predicted transcription factor binding sites based on chromatin immunoprecipitation (ChIP) studies from HEPG2 and K652 cells, falls within a GATA2 and GATA3 binding site and is bound by GATA2 in SH-SY5Y cells (ENCODE; Figure 2B and Supplementary Figure S6). *rs58722186* is also flanked by DHS peaks and enriched for histone 3 monomethylation on lysine 4 (H3K4me1) (Figure 2B and Supplementary Figure S6). *rs58722186* is one of the maximally associated serum urate variants (*p* = 7.10 × 10^-13^) in the Kanai et al. (2018) dataset. *rs58722186* is an eQTL for *MAFTRR* in multiple tissues with a similar effect on expression as the lead SUA1 urate SNP *rs7188445* (*p* = 1.3 × 10^-36^ and *p* = 9.6 × 10^-37^, respectively). Chromatin state segmentation by hidden Markov model (ChromHMM) (Ernst and Kellis, 2017) indicates that *rs58722186* is within heterochromatin (Figure 2B and Supplementary Figure S6) but is in proximity to a weak enhancer in K652 cells. We find minimal evidence for regulatory elements that overlap urate-associated SNPs identified in the European dataset in SUA1 (Supplementary Figure S2B and Supplementary Table S1) consistent with our PAINTOR analyses (Supplementary Table S3).

### The SUA2 Region Has Hallmarks of Enhancer Function

At SUA2 the lead PAINTOR SNP *rs192158533* (Table 3, Figures 2A,C, PAINTOR posterior probability = 0.89) does not mark any significant regulatory elements. *rs192158533* is monomorphic in East Asian populations yet there is a strong urate association at SUA2 signal in Kanai et al. (2018) data. The second most likely SUA2 PAINTOR SNP (Table 3, Figures 2A,C, PAINTOR posterior probability = 0.27) is *rs4077450* and the maximally associated urate SNP in the Kanai et al. (2018) Japanese data (*p* = 3.9 × 10^-14^).

ENCODE identifies that *rs4077450* along with SUA2 variant *rs4077451* encompass a region that is enriched for H3K4me1 and H3K27ac (Figure 2C and Supplementary Figure S7) (Heintzman et al., 2009). DHS peaks overlap this region in 33 cell lines, including HRCEpiC renal cortex cells (Figure 2C, Supplementary Figure S7, and Supplementary Table S4) (Thurman et al., 2012). The ENCODE data identifies ∼30 predicted transcription factor binding sites within the *rs4077450_rs4077451* SNP region in chromatin immunoprecipitation (ChIP) studies in HEPG2 and K652 cells (Figure 2C and Supplementary Figure S7) (Gerstein et al., 2012; Wang et al., 2012, 2013).

ChromHMM (Ernst and Kellis, 2017) predicts that the *rs4077450_rs4077451* SNP region marks a strong enhancer in liver carcinoma HEPG2 and leukemia K562 cells (Figure 2C and Supplementary Figure S7). In these cells there is also evidence for strong active transcription signatures at the *MAFTRR/LINC01229* TSS but the *MAF* TSS is marked by repressed chromatin signatures. Our eQTL analyses indicate that SUA2 SNPs *rs4077450* and *rs4077451* are within the colocalized *LINC01229* eQTL (*p* = 1.2 × 10^-5^ and *p* = 2.0 × 10^-5^, respectively, Supplementary Figures S8A,B) and the urate lowering alleles associate with lowered *LINC01229* expression (Table 2 and Supplementary Table S2).

Conserved non-coding regions of the genome might indicate developmental enhancers (Polychronopoulos et al., 2017). LINSIGHT (Huang et al., 2017) indicates that the SUA2 SNP region is under sequence constraint (Supplementary Figure S9). SUA2 also encompasses a HAR (Bird et al., 2007) which is located ∼2 kb upstream of the *rs4077450* SNP region at Chr16: 79934248–79934397 (Supplementary Figure S9). In combination, our *in silico* analyses indicate that the SUA2 SNP region is a strong candidate for enhancer function and follow up in functional assays.

### The SUA2 Region Functions as a Kidney-Specific Enhancer in Zebrafish

We performed transient enhancer assays in zebrafish embryos to investigate the ability of the SUA2 region to act as an enhancer *in vivo*. The region encompassing the DHS and TFBS hotspot and spanning *rs4077450* and *rs4077451* was amplified from 1000 Genome DNA samples heterozygous for each of the allele variants (Figure 2C). Allele fragments containing *rs4077450_T-rs4077451_A* and *rs4077450_G-rs4077451_T* are herein named major and minor allele fragments, respectively. The minor alleles associate with increased urate levels and increased *LINC01229* expression.

**FIGURE 3:**
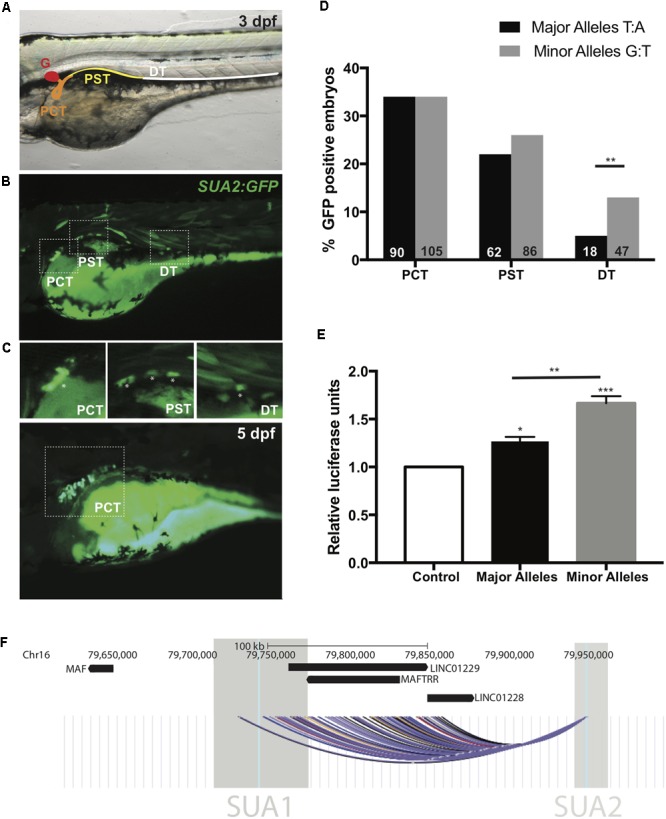
SUA2 is a functional enhancer that physically connects to the lincRNA region. An enhancer construct containing the SNP region (e.g., major alleles) injected at the one-cell stage in developing zebrafish embryos drives transient GFP reporter expression in the proximal tubules of 24 hpf, 48 hpf, and 3 dpf embryos. **(A)** 48 hpf embryo with schematic depicting the zebrafish pronephros: glomerulus (G, red); proximal convoluted tubule (PCT, orange); proximal straight tubule (PST, yellow); distal tubule (DT, green). **(B)** Representative image from a 3 dpf embryo with mosaic GFP expression in the PCT, PST and DT. Insets of the PCT, PST and DT are below. Asterisks indicate GFP positive cells. **(C)** Representative image from a 5 dpf embryo with mosaic GFP expression found exclusively in the PCT. **(D)** Percentage of transgene positive embryos that present with GFP-positive cells in the PCT, PST, and DT in enhancer constructs containing the SNP region with either the major or minor alleles for SNPs *rs4077450* and *rs4077451* at 3 dpf. The total number of embryos that were transgene-positive for the major allele fragment was 349. The total number of embryos that were transgene-positive for the minor allele fragment was 413. Numbers in each group are represented at the bottom of each bar in the graph. **(E)** Luciferase reporter assay in HEK293 cells with an empty pGL4.23 vector (control), or a pGL4.23 vector containing the SNP region with either the major (urate-lowering) or minor (urate-raising) alleles of the SNP. Luciferase expression is plotted relative to the expression of Renilla and normalized to the expression from the empty vector. A one-way ANOVA test resulted in a significant difference between the means of the control and the SNP region containing both the minor and major allele and a significant difference between the means of the major and minor alleles. Asterisks indicate the set of values that were significantly different from the control; ^∗^*p* < 0.05, ^∗∗^*p* < 0.005 and ^∗∗∗^*p* < 0.0005. **(F)** The intergenic region upstream of *MAF* that encompasses the lincRNAs *MAFTRR, LINC01229* and *LINC01228*. Also shown are the locations of SUA1 and SUA2. The region of interaction for the interaction fragment that encompasses the *rs4077450_rs4077451* SNP region is indicated, data are from Rao et al. (2014) using K562 (black), NHEK (blue), IMR90 (yellow), HMEC (red), GM12878 (purple) and KBM7 (gray) cells. The interaction region is the combination of 45 interactions with the *rs4077450_rs4077451* SNP region.

The major and minor allele fragments of the SUA2 region are both capable of driving kidney-specific GFP expression in zebrafish larvae (Figure 3). Cells that are destined to form the zebrafish pronephric duct arise from the ventral mesoderm and then populate the intermediate mesoderm where the differentiation of the pronephric duct into the glomerulus, proximal tubule and distal tubule begins (Horsfield et al., 2002; Drummond, 2003). GFP was initially observed in the zebrafish kidney proximal tubule at 24 h post-fertilization (hpf) (Supplementary Figure S10). By 3 days post-fertilization (dpf), the pronephric duct can be clearly divided into four sections; the glomerulus, the proximal convoluted tubule (PCT), the proximal straight tubule (PST) and the distal tubule (DT) (Figure 3A) (Wingert et al., 2007). At 3 dpf, the presence of mosaic GFP showed that the enhancer is active in the PCT, PST and DT (Figure 3B). By 5 dpf GFP kidney expression was confined to the PCT (Figure 3C).

The extent of GFP localization in the PCT, PST, and DT was quantified in 3 dpf embryos. The minor allele fragment drove GFP expression in the cells of the PCT in 34% of embryos, the PST in 26% of embryos and the DT in 13% of embryos at 3 dpf. The major allele fragment drove GFP expression in the PCT in 34% of embryos, PST in 22% of embryos and DT in 5% of embryos at 3 dpf (Figure 3D). The percentage of embryos with GFP in the DT between the major and minor allele fragments was significantly different (Fishers exact test, *p* = 0.0025) indicating that minor (urate-raising) allele fragment has subtly greater spatial enhancer activity during development.

### The Amplitude of SUA2 Enhancer Activity in HEK293 Cells Depends on Allele Identity

The SUA2 major and minor allele fragments were cloned into luciferase reporter constructs and evaluated for differential enhancer activity in the human embryonic kidney cell line HEK293. The major and minor allele fragments both exhibited significant (*p* = 0.0216 and *p* = 0.0002, respectively) enhancer activity in HEK293 cells when compared to the vector only control (Figure 3E). Notably, the minor urate-raising allele fragment had elevated enhancer activity when compared to the major allele (*p* = 0.0032).

Collectively, our results indicate that the genomic region containing the *rs4077450_rs4077451* SNPs is a kidney-specific regulatory element (henceforth called the SUA2 enhancer) that shows allele-specific enhancer activity. The minor (urate-raising) alleles increased the range of tissue expression in zebrafish, and also increased the amplitude of expression in HEK293 cells, providing the first indication of a mechanism for influence of SUA2-specific genetic variation on serum urate levels.

### The SUA2 Region Physically and Functionally Connects With *MAFTRR/LINC01229* and SUA1

CoDeS3D which leverages genome connectivity (Hi-C datasets) and gene expression associations [eQTL data from the GTEx catalog (Carithers and Moore, 2015)] provides evidence that variants within the SUA2 enhancer regulate the *LINC01229* locus via long-range interactions. Genome connectivity data from Hi-C datasets for K562, NHEK, IMR90, HMEC, GM12878, and KBM7 cells (Rao et al., 2014) shows that an interaction fragment containing *rs4077450* and *rs4077451* within SUA2 physically interacts with 45 restriction fragments that fall within a ∼130 kb interaction region [Figure 3F, Chr16: 79712175–79841647 (build 37.7) FDR < 0.02, Supplementary Table S5]. This interaction region includes the transcripts for the lincRNA genes *LINC01229, LINC01228*, and *MAFTRR* and a large part of SUA1 (Figure 3F). Hi-C data from renal cells (Caki-2) (Yang et al., 2018) provides further confirmation that a restriction fragment containing *rs4077450* interacts with restriction fragments containing *rs7188445* (SUA1) and *rs889472* (SUA3) (Supplementary Figure S11A). Hi-C data also shows that topologically associated domains (TADs) at the *MAF* locus shift in location depending on cell/tissue type. In NHEK cells *MAF* is found on the TAD boundary (Supplementary Figure S11B) and in HUVEC, IMR90 and KBM7 cells *MAF* shares a TAD with SUA1, *MAFTRR, LINC01229*, and SUA2 (Supplementary Figure S11B).

### The SUA2 Enhancer Binds HNF4α and Its Expression Coincides With *maf* and *HNF4A* Expression in Zebrafish Kidney

**FIGURE 4:**
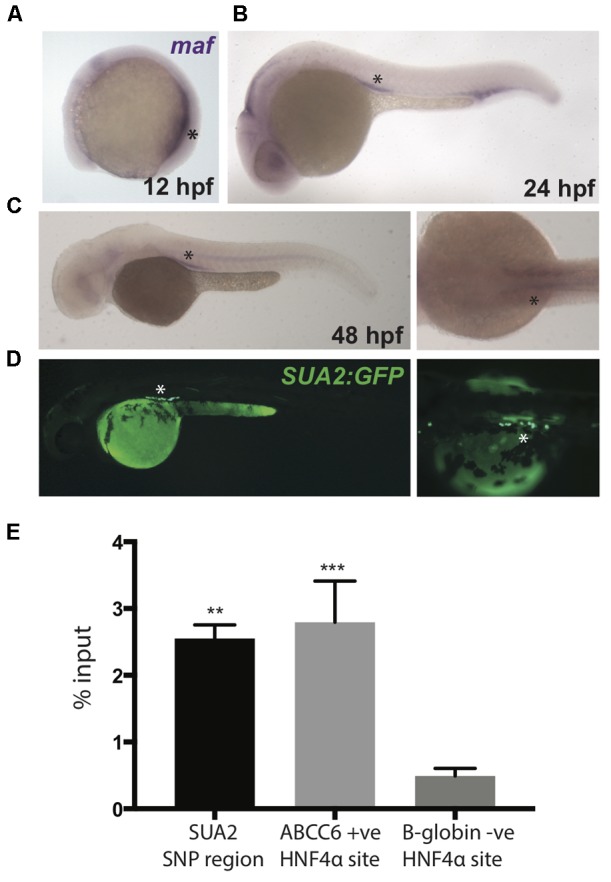
Zebrafish *maf* coincides with SUA2 enhancer activity. **(A–C)** Whole mount *in situ* hybridization with a riboprobe detecting zebrafish *maf* was performed on wild type embryos at 12, 24, and 48 hpf. *maf* expression is present in the pronephros at 12 hpf and the proximal tubules at 24 and 48 hpf. **(D)** The SUA2 SNP region drives expression of GFP in the proximal tubules at 48 hpf. **(E)** ChIP analysis of the HNF4α binding site at the SUA2 SNP region, a positive HNF4α site at *ABCC6* and a negative HNF4α site at *B-globin*. Binding is shown relative to input chromatin. Bar graphs represent mean from three independent experiments. A one-way ANOVA test indicates a significant difference between the means of the SUA2 SNP region and the negative binding site in *B-globin*. There is no difference between the positive *ABCC6* site and the SUA2 SNP region. Error bars denote standard error of the mean and asterisks indicate significance: ^∗^*p* < 0.005; ^∗∗^*p* < 0.005, ^∗∗∗^*p* < 0.0005.

We used *in situ* hybridization to examine the spatial location of *Danio rerio maf* transcripts in zebrafish embryos and assess whether *maf* expression overlaps with the activity of the kidney specific enhancer. We found that *maf* is expressed in the developing kidney and proximal tubules at 12 hpf, 24 hpf, and 48 hpf (Figures 4A–C). GFP expression driven by the SUA2 enhancer in the proximal tubules coincides with the developmental expression of zebrafish *maf* at 48 hpf (Figure 4D).

The HNF4α transcription factor is robustly expressed in the human kidney proximal tubule^2^ (Ponten et al., 2008) and regulates the expression of genes encoding proteins that control serum urate (Prestin et al., 2014), including *PDZK1* (Ketharnathan et al., 2018). Furthermore, variation at the *HNF4A* locus is associated with serum urate levels (*P*∼10^-6^) (Köttgen et al., 2013). SUA2 variants *rs4077450* and *rs4077451* flank a core HNF4α consensus motif (Supplementary Figure S7) (Wang et al., 2012; Huang et al., 2017) and fall inside an HNF4α ChIP-seq peak in HEPG2 cells (Supplementary Figure S7) (Gerstein et al., 2012; Wang et al., 2012, 2013). We conducted ChIP-qPCR using an anti-HNF4α antibody and confirmed that the SUA2 enhancer is physically bound by HNF4α in HEPG2 cells (Figure 4E). HNF4α binding was not assessed in HEK293 cells because *HNF4A* is not expressed in this cell line (Ketharnathan et al., 2018). GFP expression driven by the SUA2 enhancer also coincides with *hnf4a* expression in the zebrafish pronephric duct at these stages (Wingert and Davidson, 2011). Combined, our analyses of the SUA2 kidney enhancer indicate that it functionally and physically connects with the *MAFTRR/LINC01229* lincRNA region, is likely responsive to HNF4α, and could contribute to *MAF* expression. However, several other transcription factor consensus motifs, including those for TFAP2A and HNF4G, are also in close proximity to SUA2 variants *rs4077450* and *rs4077451*, therefore it is possible that the variants could influence the binding of one or more transcription factors.

### SUA1 Variants in *MAFTRR* and *LINC01229* Have Targets Independent of *MAF* That Influence Serum Urate Levels

CoDeS3D and Hi-C data for K562, NHEK, IMR90, HMEC, GM12878 and KBM7 cells provides spatial data supporting the *rs7188445 MAFTRR* cis-eQTL (Supplementary Table S6). Hi-C data also provide evidence that the lead *MAFTRR* eQTL SNPs which lie within the *MAFTRR* and *LINC01229* transcripts physically connect with *MAF*, however, there are no significant *MAF* eQTLs associated with these connections (Supplementary Table S7).

**FIGURE 5:**
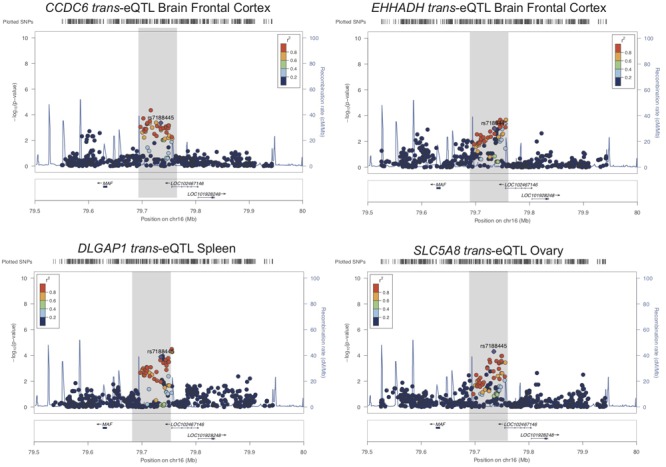
SUA1 connects with and associates with expression of genes in *trans.* Regional association plots for four *trans*-eQTL at SUA1 implicated in serum urate and kidney relevant pathways. Dots indicate individual SNPs while their position relative to the left *Y*-axis indicates significance [-log_10_(*p*-value)] of association to serum urate levels. The blue line indicates the recombination rate across the locus. The SUA1 region upstream of *MAF* is indicated by a gray box. *rs7188445* (the lead SUA1 urate SNP) is indicated by a purple dot. The color of the surrounding SNPs indicates the strength of LD with *rs7188445* according to the key in the left top hand corner, measured as *r^2^* found in the HapMap data (hg19/1000 genomes Nov 2014) for Europeans. The plot was generated using LocusZoom (Pruim et al., 2010).

We also tested whether genes in *trans* have functional genomic regulatory connections at SUA1 (Rinn and Chang, 2012). CoDeS3D identified 12 spatially supported *trans*-eQTL with SUA1 variants located within the *MAFTRR* and *LINC0122*9 transcripts (FDR < 0.05, Supplementary Table S8) and also with the lead SUA1 SNP *rs7188445* (Supplementary Table S6). The lead SNPs for these *trans*-eQTL at SUA1 are different than the lead *cis*-eQTL SNPs for *MAFTRR* (Supplementary Figures S2, S12 and Figure 5). Importantly COLOC analysis (Giambartolomei et al., 2014) indicates that these *trans*-eQTL signals colocalize with the SUA1 serum urate signal (Table 4) indicating that SUA1 could control serum urate levels via *trans-*eQTL connections. Of the 12 *trans-*eQTL, only 2 did not colocalize (H4 < 0.5) with the SUA1 signal (*CCDC79* and *TBC1D10B*). Of the 10 remaining *trans-*eQTL, four genes (i.e., *SLC5A8, CCDC6, EHHADH*, and *DLGAP1*) are potentially involved in urate metabolism based on our Gene Ontology (GO) analyses^3^ and previous urate associations (Köttgen et al., 2013; Kanai et al., 2018) (Figure 5).

**Table 4:** Colocalization of Köttgen SUA1 signal and SUA1 *trans*-eQTL signals.

Locus	PPC^1^	eQTL tissue
*SLC5A8*	0.80	Ovary
*TNKS*	0.93	Heart left ventricle
*EHHADH*	0.69	Brain frontal cortex
*ENTPD3-AS1*	0.85	Stomach
*ARHGAP35*	0.68	Uterus
*CCDC6*	0.63	Brain frontal cortex
*RP11-386L12.1*	0.68	Brain caudate basal ganglia
*SLCO3A1*	0.52	Brain hippocampus
*BTLA*	0.58	Uterus
*CCDC79*	0.25	Breast mammary tissue
*DLGAP1*	0.78	Spleen
*TBC1D10B*	0.09	Colon sigmoid

*^*1*^Posterior probability of colocalization (PPC) as determined by COLOC.*

*CCDC6* is in very close proximity to a urate GWAS signal that sits in the intergenic region between *CCDC6* and *SLC16A9* (Supplementary Figure S13A). *CCDC6* and *SLC16A9* are strongly co-regulated (*p* = 6.9 × 10^-9^, GeneNetwork.nl) and the lead Köttgen SNP at *CCDC6/SLC16A9* (*rs1171614*) is an eQTL for both *SLC16A9* and *CCDC6* [GTEx (Carithers and Moore, 2015)]. SUA1 lead urate-raising variant *rs7188445_G* associates with lowered expression of *CCDC6* (Supplementary Figure S13B). At *SLC5A8* there is a weak urate GWAS signal in the Japanese population (Kanai et al., 2018) (Supplementary Figure S13A). *SLC5A8* is a monocarboxylate transporter that has been strongly implicated in urate transport in the kidney (Merriman, 2015) and associates with the GO biological function term urate transport (*p* = 6.3 × 10^-3^) (Supplementary Table S9). The urate-raising allele of *rs7188445* associates with increased expression of *SLC5A8* (Supplementary Figure S13B). The only other genes with a *trans*-eQTL at SUA1 with evidence for a urate signal was *DLGAP1* in the Japanese population (Supplementary Figure S13A). The serum urate-raising and gout risk allele *rs7188445_G* associates with increased expression of *DLGAP1* (Supplementary Figure S13B). The proteins encoded by both *DLGAP1* and *SLC5A8* contain PDZ3 domains and interact with PDZK1 (Hu et al., 2009), a scaffold protein involved in urate reabsorption within the kidney tubule (Merriman, 2015).

Of the 10 genes with colocalized SUA1 *trans*-eQTL, *EHHADH* has the strongest signal in kidney tubule (gene level FDR = 0.078) (Gillies et al., 2018). Expression of *EHHADH* is enriched in the proximal tubule (Habuka et al., 2014) and associates with the GO biological function term urate transport (*p* = 7.7 × 10^-3^) in the GeneNetwork database (Supplementary Table S9). The serum urate-raising and gout risk allele *rs7188445_G* associates with increased *MAFTRR* expression and lowered *EHHADH* expression (Supplementary Figure S13B). These analyses collectively indicate that SUA1 variants associate with the expression of serum urate and kidney relevant genes in *trans*.

### *LINC01229, MAFTRR*, and *Trans*-eQTL Are Coexpressed With *MAF*

Our analyses indicate that *LINC01229* and *MAFTRR*, along with genes in *trans*, likely have functions in serum urate regulation. In addition, the SUA2 enhancer recruits HNF4α and coincides with the expression of zebrafish *hnf4a* and *maf*, implicating *MAF* in serum urate regulation. Supporting this co-expression analysis the GeneNetwork database (Kumar et al., 2013) indicates that *MAFTRR* and *LINC01229* are strongly co-expressed with *MAF* (*p* = 5.0 × 10^-27^ and *p* = 2.4 × 10^-26^, respectively). However, *MAFTRR* and *LINC01229* are not co-expressed (Supplementary Tables S10, S11). Interestingly, *HNF4A* is co-expressed with *LINC01229* (*p* = 0.011) but not with *MAFTRR* (Supplementary Tables S10, S11) consistent with the SUA2 variant effect on *LINC01229* expression. We also assessed whether the SUA1 *trans*-eQTL genes are co-expressed with *MAFTRR, MAF* and/or *LINC01229*. *SLCO3A1, TNKS* and *ENTPD3* (the likely target of *ENTPD3-AS1*) are co-regulated with *MAF* (*p* = 0.0088, *p* = 0.011, and *p* = 0.022, respectively) (Supplementary Table S12) and *rs7188445_G* associates with increased *SLCO3A1, TNKS*, and *ENTPD3* expression (Supplementary Figure S13B).

### *MAFTRR* and *LINC01229* Regulate *MAF* and Function in Kidney and Urate Relevant Pathways

**FIGURE 6:**
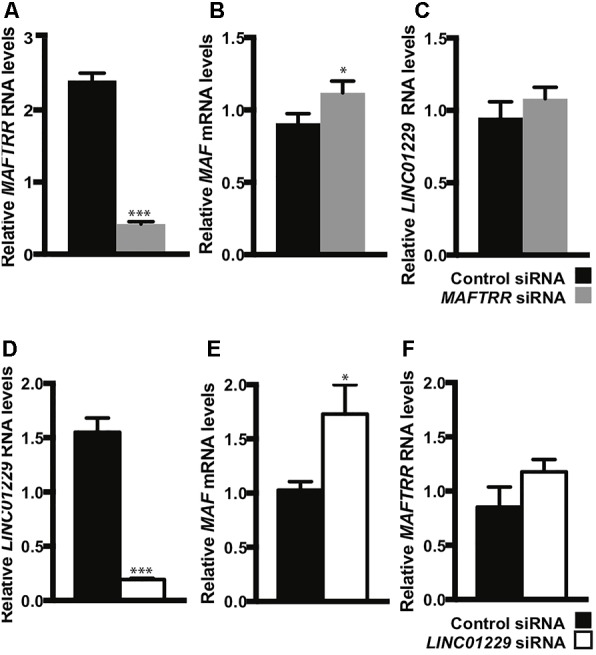
Depletion of lincRNAs increases *MAF* mRNA levels in HEK293 cells. HEK293 cells were reverse-transfected with a control siRNA, 5 nM *MAFTRR* siRNA **(A–C)** or 5 nM *LINC01229* siRNA **(D–F)**, and quantitative PCR was performed on RNA collected at 48 h post-transfection. **(A)** *MAFTRR* RNA transcript levels. **(B)** *MAF* transcript levels. **(C)** *LINC01229* RNA transcript levels. **(D)** *LINC01229* RNA transcript levels. **(E)** *MAF* RNA transcript levels. **(F)** *MAFTRR* transcript levels. Data represent four biological replicates; statistical significance was determined using unpaired *t*-test with one tail. Error bars denote standard error of the mean and asterisks indicate significance: ^∗^*p* < 0.05; ^∗∗∗^*p* < 0.005.

To test if *MAFTRR* and *LINC01229* can contribute to the regulation of *MAF* transcription, we depleted *MAFTRR* and *LINC01229* using siRNA in HEK293 cells. Reduction of *MAFTRR* RNA levels (Figure 6A) resulted in upregulation of endogenous *MAF* mRNA (Figure 6B). Reduction of *LINC01229* RNA levels (Figure 6D) also led to upregulation of endogenous *MAF* mRNA (Figure 6E). Our results indicate that *LINC01229* and *MAFTRR* normally repress *MAF* expression in kidney HEK293 cells.

Our analyses indicate that *LINC01229* and *MAFTRR* have the ability to regulate *MAF* in kidney cells. We assigned putative phenotypes and biological processes to *LINC01229* and *MAFTRR* using GeneNetwork (Kumar et al., 2013), which infers functional enrichment from co-expressed genes^4^. The top enriched human phenotype ontology terms for *MAFTRR* and *LINC01229* are ‘tubulointerstitial abnormality’ (*p* = 3.9 × 10^-4^) and ‘accelerated skeletal maturation’ (*p* = 4.3 × 10^-5^), respectively. The top GO biological process term for *MAFTRR* is *‘*chondroitin sulfate catabolic process’ (*p* = 8.4 × 10^-3^). The top GO biological process term for *LINC01229* ‘cell development’ (*p* = 7.9 × 10^-5^) implicates *LINC01229* in a pathway with *MAF* and three other serum urate-associated loci, *INHBB, INHBC* and *INHBE*, detected by GWAS (Köttgen et al., 2013) (Supplementary Figures S14A,B). Additionally, *HNF4A* and *LINC01229* share GO enrichment in lipid-related pathways including ‘chylomicron assembly,’ ‘phospholipid efflux,’ and ‘high-density lipoprotein assembly’ (Supplementary Figure S14A). *LINC01229* also associates with inflammation- and gout-relevant GO terms including ‘interleukin β secretion,’ ‘T-cell differentiation,’ and ‘interleukin 2 biosynthetic process’ (Supplementary Figure S14A).

### The *MAF* Upstream Intergenic Region Is Conserved

The intergenic region upstream of *MAF* encompassing the lincRNAs is conserved in mammals. Two non-coding RNA loci upstream of *MAF* in mouse (i.e., Gm30925 and Gm31037) are positionally conserved with respect to *MAF*, expressed in kidney and homologous with the human lincRNA region (BLAST E-value = 3 × 10^-59^ and 2 × 10^-16^, respectively) (Supplementary Figure S15). Moreover, sequence homologous (E-value = 6 × 10^-20^) to the SUA2 enhancer in mouse is located 276,670 bp upstream of *Maf*. Our analyses suggest that the ∼300 kb *MAF* upstream intergenic region marked by SUA1 and SUA2 represents an evolutionarily conserved regulatory network active in kidney and serum urate regulation.

## Discussion

**FIGURE 7:**
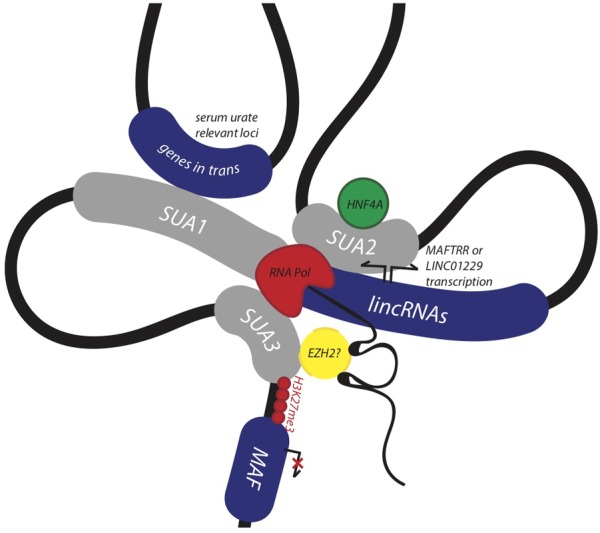
Non-coding urate-associated variants in SUA1 and SUA2 function in a conserved genomic regulatory domain with kidney and serum urate relevant functions. The schematic depicts the proposed 3D structure at the *MAF* locus and upstream region encompassing SUA1, SUA2, and SUA3 as determined by Hi-C interaction data from the kidney cell line Caki2. The lincRNAs *MAFTRR* and *LINC01229* influence expression of *MAF* in kidney, while serum urate-associated variants within the SUA2 kidney tubule enhancer physically connect with the lincRNA region, SUA1 and SUA3. SUA2 is bound by HNF4α and SUA2 urate-raising variants increase the expression of *LINC01229*. SUA1 is a spatially supported eQTL for *MAFTRR* which functions in the kidney tubule and genes *in trans* with serum urate relevant functions.

Identifying and assigning function to genomic regions encompassing disease-associated variants is crucial for identifying their biological role in disease (Edwards et al., 2013). Here, we provide evidence that the conserved syntenic block encompassing *MAF*, the lincRNA eQTL SUA1 region, and the enhancer SUA2 region constitute a functional genomic regulatory domain that contributes to serum urate regulation (Figure 7). SUA1 and SUA2 are independent and functionally distinct serum urate-associated regions that physically connect with the lincRNA region and the *MAF* promoter region (SUA3) (Figure 7). Genetic variation within SUA1 and SUA2 is associated with alterations in the expression of the lincRNAs contained in this genomic block. SUA1 variants physically connect with, and alter the expression of, *MAFTRR, LINC01229* and urate-relevant genes in *trans.* SUA2 marks an enhancer element that physically and functionally connects with *LINC01229* expression, recruits HNF4α, and coincides with the developmental expression of zebrafish *maf* and *hnf4a.* Finally, we show that the lincRNAs regulate the expression of *MAF* in *cis.* By teasing out the molecular mechanisms that underlie this regulatory network, we have assigned serum urate-associated variants to a distinct biological pathway that is active in the kidney and involved in serum urate control.

Serum urate excretion is predominantly carried out in the kidney tubules with the remainder managed by the intestine (Merriman, 2015). We observed a colocalization of the SUA1 urate signal and the *MAFTRR* colon *cis*-eQTL (Table 2 and Supplementary Figure S2) and the partial overlap with the *MAFTRR* kidney tubule eQTL signal (Supplementary Figure S2). This is consistent with our co-expression analyses which indicate that *MAFTRR* associates with the kidney-relevant human phenotype ontology term ‘tubulointerstitial abnormality.’ Collectively these data support a biological role for *MAFTRR* in serum urate regulation in these tissues.

An inverse relationship between *MAFTRR* levels and *MAF* expression has previously been identified in patients with multiple sclerosis, whereby higher levels of *MAFTRR* in CD4+ T lymphocyte cells lowers expression of *MAF* (Zhang et al., 2017). This relationship shows a strong correlation with annual relapse rates (Zhang et al., 2017). In our study, the urate-raising alleles in SUA1 increase *MAFTRR* expression (Table 2 and Supplementary Table S1). Chromosome conformation capture data in T-lymphocytes has identified genomic contacts in the region 3′ to *MAFTRR* that overlap SUA1 and physically connect with the *MAF* promoter marked by SUA3. In this genomic conformation the *MAFTRR* transcripts act as a scaffold for regulatory elements including EZH2 that recruit the repressive histone mark, H3K27me3 (Ranzani et al., 2015) which in turn represses *MAF* transcription. Our analyses of Hi-C data support this physical connection between SUA1 variants and *MAF* (Supplementary Table S7). We also observed that depleting *MAFTRR* or *LINC01229* increases *MAF* transcription in kidney cells (Figure 6), consistent with this chromatin interaction data from T-cells. We propose that the SUA1 variant effect on *MAFTRR* and/or *LINC01229* expression may also contribute to the 3D structure at this locus in a tissue-dependent manner, altering the expression of *MAF* in *cis* (Figure 7).

We also observed that SUA1 variants form functional *trans* chromatin contacts that colocalize with the SUA1 urate signal (Table 4 and Figures 5, 7). We identified *SLC5A8* which encodes sodium-coupled monocarboxylate transporter 1 (SMCT1). *SLC5A8*/SMCT1 transports monocarboxylates into the kidney tubule, which are exchanged for urate by urate reuptake transporters such as SLC22A12/URAT1 (Merriman, 2015). The urate-raising allele of *rs7188445* is associated with increased expression of *SLC5A8*, which would be predicted to increase the monocarboxylate pool for reabsorption of urate. Any serum urate signal at the *SLC5A8* locus, if real, is weak (Supplementary Figure S13); nevertheless, our data suggest that the expression of *SLC5A8* is controlled from the *MAF* SUA1 locus. *EHHADH* (associated with the GO term ‘urate transport’) is involved in fatty acid oxidation, the main energy source for kidney tubule cells (Balaban and Mandel, 1988) and *EHHADH* mutations have been implicated in Fanconi’s syndrome which is characterized by renal proximal tubule dysfunction (Klootwijk et al., 2014). Furthermore Fanconi’s syndrome can result in hypouricemia due to high urate clearance (Meisel and Diamond, 1977). A reduction in *EHHADH* expression has been observed in mouse models for chronic kidney disease (Trudu et al., 2017), hyperlipidemia (Mirzoyan et al., 2017), obesity and fatty liver disease (Vandanmagsar et al., 2011). This is consistent with the effect seen for serum urate-raising variant *rs7188445_G* at SUA1, which associates with lowered *EHHADH* expression.

We also find that a subset of genes with spatial *trans*-eQTL at SUA1 are co-regulated with *MAF*. *TNKS* and *SLCO3A1* are co-regulated with *MAF*, and expressed in kidney (Tamai et al., 2000; Karner et al., 2010). *SLCO3A1* mediates the transport of prostaglandins, thyroxin and vasopressin (Huber et al., 2007) and *TNKS* is essential for kidney development (Karner et al., 2010). *ENTPD3-AS1*, likely regulates *ENTPD3* (co-regulated with *MAF*), which is involved in purine metabolism (Kanehisa et al., 2017) of which urate is a by-product.

SUA2, the second independent region of association upstream of *MAF*, has previously been validated as a functional enhancer by high-throughput massively parallel reporter assays in HEPG2 cells (Kheradpour et al., 2013). Our study confirms that this region represents a conserved enhancer for tissue specific expression in the kidney tubule (Figure 3). A 562-bp DNA fragment containing the *rs4077450_rs4077451* SNP region within SUA2 is sufficient to confer kidney tubule expression that coincides with expression of zebrafish *maf* (Figure 4), matches the tissue expression profile of *MAF* in human and is consistent with the role MAF plays in kidney tubule development (Imaki et al., 2004). We show that the urate-raising minor alleles also increase enhancer activity in kidney cell lines (Figure 3). Our results indicate that the serum urate-associated SNPs at this region are functional and alter the function of the SUA2 enhancer, consistent with our PAINTOR analyses (Table 3).

The underlying mechanism for enhancer function has been suggested to involve the formation of long-range chromatin loops (Denker and de Laat, 2016) that bring enhancers and promoters into spatial proximity (Dixon et al., 2012). Our analysis of publicly available Hi-C data (Rao et al., 2014) indicates that the SUA2 kidney enhancer element physically interacts with the neighboring lincRNA region, including *LINC01229, MAFTRR* and part of SUA1 (Figure 3). Hi-C data from renal cells supports this interaction, in addition to a further connection between SUA2 and the *MAF* promoter (SUA3) (Supplementary Figure S11). Our eQTL analyses indicate that the SUA2 genomic contacts with *LINC01229* are functional (Fadason et al., 2017), colocalize with the SUA2 urate signal (Table 2) and that the serum urate-raising alleles of *rs4077450* and *rs4077451* increase the expression of *LINC01229* but not of *MAFTRR* (Supplementary Figure S8).

Because the SUA2 human enhancer element can drive gene expression in zebrafish kidney, this genomic region must contain highly conserved kidney-responsive regulatory elements. *rs4077450* and *rs4077451* flank an HNF4α site which has previously been shown to be required for the enhancer activity of the region (Kheradpour et al., 2013). We confirmed that HNF4α binds to this locus in HEPG2 cells (Figure 4E). Previous observations in zebrafish found that *hnfa* is expressed in the zebrafish proximal tubule in the same cell types and stages in which the SUA2 enhancer is active (Wingert and Davidson, 2011). Therefore, HNF4α is a likely candidate for driving kidney-specific activity of this enhancer. This is consistent with our understanding of (1) the role HNF4α plays in the development of the human kidney tubule (Martovetsky et al., 2013); and (2) the association of *HNF4A* with serum urate (Köttgen et al., 2013; Ketharnathan et al., 2018). Our co-expression analysis also indicates that *HNF4A*, is co-expressed with *LINC01229* but not *MAFTRR*, consistent with the SUA2 *LINC01229* eQTL and the coincidental expression of the SUA2 enhancer and *hnf4a*. *LINC01229* and *HNF4A* also share enriched GO terms in lipid relevant pathways that involve genes with (albeit unreplicated) reports of association with urate (e.g., *ApoE*) (Sun et al., 2015) and gout (e.g., *ABCG1* and *ABCA1*) (Lai et al., 2012) (Supplementary Figure S14).

While the eQTL signals for *MAFTRR* in all GTEx tissues are confined to the same region that generally overlaps SUA1, the genomic locations of the eQTL signals for *LINC01229* are diverse and tissue dependent (Supplementary Figure S2). We propose that while *MAFTRR* expression is controlled in a similar manner in a range of tissues, *LINC01229* expression is under the control of tissue-specific and perhaps development-specific regulatory elements that are distributed throughout the lincRNA locus. The SUA2 region is evidence of one such region: (1) it contains an enhancer element that drives expression exclusively in the developing zebrafish kidney tubule; and (2) it contains SUA genetic variants that have the ability to alter *LINC01229* expression. We propose that in kidney, long-range chromatin interactions bring the HNF4α-responsive SUA2 enhancer into close spatial proximity with the SUA1 lincRNA region and the SUA3 *MAF* promoter region, implicating *LINC01229* as a likely regulatory target of the SUA2 enhancer. We predict that this chromosome conformation (Figure 7) would have consequences for *MAF* expression, as evidenced by the loss of *MAF* repression following siRNA knockdown of *LINC01229* in kidney cells (Figure 6), and consistent with the upregulation of *maf* observed in *hnf4a* mutant zebrafish (Davison et al., 2017). Our GO analyses indicate that *LINC01229* could function in a cell development pathway with *MAF* and the inhibin genes *INHBC, INHBA* and *INHBB* previously associated with urate (Köttgen et al., 2013). *LINC01229* and *MAF* could mediate serum urate homeostasis through known inhibin pathways such as apoptosis (Denkova et al., 2004) and inflammation (Okuma et al., 2005) consistent with our GO analyses, which identified ‘negative regulation of cell apoptotic process’ and ‘interleukin 1 β secretion’ as enriched terms for *LINC01229*. In addition to this ‘interleukin 1 β secretion’ is strongly implicated in gout (So and Martinon, 2017).

The multiplicity of phenotype associations at the lincRNA locus suggests that variants at this region and their effect on *MAFTRR, MAF* and/or *LINC01229* could also be biologically important in other disease pathways (Table 1 and Supplementary Figure S1). Serum urate levels have previously been correlated with multiple sclerosis (Wang et al., 2016), hyperthyroidism (Giordano et al., 2001; See et al., 2014), obesity (Tanaka et al., 2015), fatty liver disease and chronic kidney disease and the SUA regions upstream of *MAF* coincide with GWAS signals of these conditions. SUA1 coincides with liver- and thyroid-related traits whereas SUA2 coincides with kidney related traits (Supplementary Figure S1). *MAF* expression has also been associated with multiple sclerosis (Zhang et al., 2017), a range of cancers including renal cancer (Ponten et al., 2008), glucagon expression and biosynthesis (Planque et al., 2001; Kataoka et al., 2004; Gosmain et al., 2007) and autoimmune diabetes (Pauza et al., 2001). Thus, the shared association of these diseases at the lincRNA region may provide a causal link to serum urate and/or the expression of the lincRNAs and *MAF*.

In our analyses, we have identified a program of genes that can be modulated by variants within SUA1 and SUA2 and are likely important in kidney tubule development and function and serum urate regulation. We conclude that tissue-specific and developmental control of the lincRNA region appears to be important for the modulation of *MAF* and other urate relevant gene pathways in *trans*.

## Materials and Methods

### Genetic Analyses

Publicly available summary level statistics from the Köttgen et al. (2013) GWAS for serum urate were used. Conditional association analysis on *rs7188445* was performed using the Genome-Wide Complex Trait Analysis (GCTA) software (Yang et al., 2011). 6,654 HapMap2 imputed genotypes from European participants of the Atherosclerosis Risk in Communities study were used as a reference. LD *R*^2^ values were calculated using LDlink (Machiela and Chanock, 2015) with the 1000 Genomes European and East Asian reference panels.

### PAINTOR Analysis

ImpG (v1.0) (Pasaniuc et al., 2014) was used to impute Z-scores into the European urate GWAS (Köttgen et al., 2013) summary statistics. For the reference haplotypes, the latest release of the 1000 Genomes project was used, and only bi-allelic SNP markers having a minor allele frequency greater than 0.01 in the relevant population were included. All imputed markers with a predicted LD *R*^2^ of less than 0.8 were removed. PAINTOR leverages functional genomic annotation data, in addition to genetic association strength, to prioritize functional variants. The LD matrix used for fine mapping of the Köttgen GWAS data in PAINTOR was calculated based on European data from the 1000 Genomes Project Phase 1. The annotation matrix used in PAINTOR was kidney cell-type DHS (Finucane et al., 2015). PAINTOR (v3.0) (Kichaev et al., 2014) was carried out using a LD matrix matched locus file for SUA1 and SUA2.

### COLOC Analysis

We used COLOC (Giambartolomei et al., 2014) to assess the similarity between the urate-associated loci SUA1 and SUA2 and publicly available eQTL data from the Genotype Tissue Expression Project (GTEx). COLOC is a Bayesian method that compares four different statistical models at a locus and gives a posterior probability for each test. The four tests are: H0: no causal variant in the GWAS or an eQTL region H1: a casual variant in either the GWAS or the eQTL region but not both, H3: a causal variant in the GWAS and the eQTL region that is different, H4: a causal variant in the GWAS and the eQTL region that is shared.

### Integration of GWAS and eQTL Results With CoDeS3D

The Contextualize Developmental SNPs using 3D Information (CoDeS3D) algorithm (GitHub^5^) (Fadason et al., 2017) was used to identify long-distance regulatory relationships for *rs4077450* and *rs4077451* (SUA2), *rs7188445* (SUA1) and SUA1 SNPs that overlap the *MAFTRR* transcript. This analysis leverages known spatial associations from Hi-C databases (Rao et al., 2014) and gene expression associations [eQTL data from the GTEx catalog (GTEx 2013)] to assess regulatory connections. Briefly, SNPs were mapped onto Hi-C restriction fragments, the genes that physically interact with these restriction fragments identified and collated (SNP-gene spatial pairs). SNP-gene pairs were screened through GTEx to identify eQTLs. The false discovery rate (FDR) was calculated using a stepwise Benjamini-Hochberg correction procedure and incorporated the number of tests and eQTL value list. A FDR value of <0.05 was accepted as statistically significant (Fadason et al., 2017).

### Vectors and Constructs

A DNA fragment (562 bp) harboring *rs4077450_rs4077451* (Chr16: 79931421–79931980) was amplified from the genomic DNA of individuals who were heterozygous for the major alleles (*rs4077450_T-rs4077451_A)* and the minor alleles (*rs4077450_G-rs4077451_T*), respectively. Primers F: 5′-CCTCCATACAGTGTCCAGCA-3′ and R: 5′-TGGACCGTTTTGGCTTTTAC-3′ were used for amplification. Amplicons were cloned into the pCR^®^8/GW/TOPO (Invitrogen) entry vector and gateway-cloned into the destination vectors pGL4.23 (addgene 60323) and ZED (Bessa et al., 2009) using LR Clonase^®^ enzyme (Invitrogen) for the enhancer assays in human cell lines and zebrafish, respectively.

### Cell Culture and Transfections

Human embryonic kidney HEK293 cells (ATCC) and human hepatocellular carcinoma HEPG2 cells (ATCC) were grown (37°C in a 5% CO_2_) and maintained in Dulbecco’s Modified Eagle’s Medium (DMEM) and Eagle’s Minimum Essential Media (EMEM), respectively, (Life Technologies), supplemented with 10% fetal bovine serum.

For luciferase assays, HEK293 cells were transiently transfected with pGL4.23 constructs and Renilla, using Lipofectamine 3000 (Invitrogen). Luminescence was measured 48 h post-transfection using the Dual Glo Luciferase Assay (Promega), on the Perkin Elmer Victor X4 plate reader. Values were normalized to the expression of Renilla luciferase.

For the siRNA knockdown experiments, HEK293 cells were reverse-transfected with ON-TARGETplus Non-targeting Control Pool siRNA (5 nM) (Dharmacon Cat# D-001810-10), Lincode SMARTpool *LINC01229* siRNA (5 nM) (Dharmacon Cat# R-191862-00-0005), and Lincode SMARTpool *MAFTRR* (5 nM) (Dharmacon Cat# R-192817-00-0005) using RNAiMax (Invitrogen) for gene expression analysis.

### Quantitative PCR

Total RNA was isolated from control and siRNA-treated HEK293 cells at 48 h post-treatment using the NucleoSpin RNA kit (Macherey-Nagel). cDNA was synthesized with qScript cDNA SuperMix (Quanta Biosciences). *MAF, MAFTRR* and *LINC01229* expression were measured using TaKaRa SYBR Premix Ex Taq^TM^ (Clontech) on a LightCycler400 (Roche Diagnostics). Primers *MAF* F: 5′-AGCAAGTCGACCTCAAG-3′ and R: 5′-CGAGTGGGCTCAGTTATGAAA-3′, *MAFTRR* F: 5′-CCTGGACAATGCTGGTTTTT-3′ and R: 5′-CACGTCCTTCCATTTTGCTT-3′ and *LINC01229* F: 5′-ATGGGAGCTCCACACAGGT-3′ and R: 5′-TGGGTGCCTTTAAACAAGAGA-3′ were used for amplification. Gene expression analyses were carried out on qBase Plus (Biogazelle) and were normalized relative to the mean of reference genes encoding glyceraldehyde 3-phosphate dehydrogenase (*GAPDH*) and ribosomal protein L13a (*RPL13A*).

### Quantitative Chromatin Immunoprecipitation

Chromatin was extracted from 10^-7^ HEPG2 cells, sonicated (Vibra Cell VCX130 Sonicator, Sonics) to approximately 500 bp (determined experimentally) and diluted 10 times with immunoprecipitation (IP) buffer as described previously (Veto et al., 2017). Chromatin immunoprecipitation (ChIP) was carried out according to Vaisanen et al. (2005) with minor adjustments. Equal amounts of diluted chromatin in 2 ml IP buffer were pre-cleared (to reduce background) overnight with Dynabeads protein G (Thermo Fisher). Immunoprecipitations were performed overnight at 4°C with 10 μl of a ChIP grade HNF4α (ab41898, Abcam) antibody conjugated to 50 μl of Dynabeads G. The immunocomplexes were then pelleted by centrifugation at 4°C for 1 min at 100 × *g* and washed sequentially for 5 min by rotation with 1 ml of the following buffers: low-salt wash buffer [20 mM Tris–HCl (pH 8.1), 0.1% SDS, 1% Triton X-100, 2 mM EDTA, 150 mM NaCl], high-salt wash buffer [20 mM Tris–HCl (pH 8.1), 0.1% SDS, 1% Triton X-100, 2 mM EDTA, 500 mM NaCl] and LiCl wash buffer [10 mM Tris–HCl (pH 8.1), 0.25 mM LiCl, 1% (v/v) Nonidet P-40, 1% (w/v) sodium deoxycholate, 1 mM EDTA]. Finally, the beads were washed twice with 1 ml of TE buffer [10 mM Tris–HCl (pH 8.0), 1 mM EDTA] and eluted in ChIP elution buffer (50 mM Tris, pH 8, 10 mM EDTA, 1% SDS) at room temperature for 30 min with rotation. Crosslinking was reversed with Proteinase K (final concentration 40 μg/ml) at 50°C for 2 h, DNA was recovered using phenol:chloroform:isoamyl alcohol (Invitrogen) and precipitated with 0.1 volume of NaCl and 2.5 volumes of ethanol using Ambion^®^ Linear Acrylamide (5 mg/mL) as a carrier. For qPCR analyses, 1 μl of pre-cleared or immunoprecipitated chromatin was used for each reaction. HNF4α binding at the SNP region, negative binding site B-globin and positive binding site ABCC6 (Veto et al., 2017) was expressed relative to the pre-cleared input chromatin after subtraction from no antibody control. Primers SNP region F: 5′-CCTCCATACAGTGTCCAGCA-3′ and R: 5′-TGGACCGTTTTGGCTTTTAC-3′, B-globin F: 5′-AGGACAGGTACGGCTGTCATC-3′ and R: 5′-TTTATGCCCAGCCCTGGCTC-3′ and ABCC6 F: 5′-AGCCCATTGCATAATCTTCTAAGT-3′ and R: 5′-ATGGAGACCGCGTCACAG-3′ were used for amplification.

### Zebrafish Enhancer Assays

Wild type (WIK) fish lines were maintained according to established protocols (Westerfield, 2000). The University of Otago Animal Ethics Committee approved all zebrafish research.

An enhancer test vector ZED (Bessa et al., 2009) was used to investigate the enhancer capacity of the genomic region (Chr16: 79931421–79931980) marked by *rs4077450* and *rs4077451*. Single-cell WIK embryos were injected with 1 nl of 30 ng/μl ZED (Bessa et al., 2009) DNA construct containing either of the minor and major fragments for the *rs4077450_rs4077451* SNP region and 90 ng/μl Tol2 transposase mRNA (Kawakami, 2005). Injected embryos were screened for Green Fluorescent Protein (GFP) expression 24–120 h post-fertilization (hpf) using a Leica M205 FA fluorescence microscope.

### Whole Mount *in situ* Hybridization

Full length *maf* riboprobe synthesis and whole mount *in situ* hybridization was performed as described previously (Monnich et al., 2009). The cDNA of *MAF* on a pCMV SPORT 6.1 plasmid was acquired from Life Biosciences as the *Danio rerio maf* cDNA DR.81288. The plasmid was linearized with *Apa*I and the antisense DIG-labeled riboprobe was generated by *in vitro* transcription using SP6 RNA polymerase (Roche).

### Statistical Analysis

GraphPad PRISM 7 (GraphPad software, San Diego, CA, United States) was used for performing all statistical analysis. One-way ANOVA, (Tukey’s multiple comparisons tests), Fisher exact tests and unpaired *t*-tests were used for estimating the statistical significance of the luciferase, enhancer assays and quantitative PCR data, respectively. All data are presented as mean and error bars represent standard error of the mean (SEM).

## Author Contributions

ML, JH, JO, and TM designed the study. ML performed the functional annotations, the eQTL analyses, the enhancer assays, the *in situ* hybridization, and the siRNA assays. HS isolated and cloned the *rs4077450_rs4077451* minor and major allele fragments into the plasmid constructs and was supervised by JM. AD carried out the luciferase assay and was supervised by JA. RT carried out the conditional analysis. WS and TF carried out the CoDeS3D analyses. ML, JH, and TM wrote the manuscript with input from JO and WW.

## Conflict of Interest Statement

The authors declare that the research was conducted in the absence of any commercial or financial relationships that could be construed as a potential conflict of interest.

## Footnotes

1 http://jenger.riken.jp:8080

2 https://www.proteinatlas.org/

3 www.GeneNetwork.nl

4 www.genenetwork.nl/genenetwork

5 https://github.com/Genome3d/codes3d-v1
