# Corrigendum: Functional Urate-Associated Genetic Variants Influence Expression of lincRNAs *LINC01229* and *MAFTRR*

**DOI:** 10.3389/fgene.2019.00382

**Published:** 2019-04-24

**Authors:** Megan Leask, Amy Dowdle, Hamish Salvesen, Ruth Topless, Tayaza Fadason, Wenhua Wei, William Schierding, Judith Marsman, Jisha Antony, Justin M. O'Sullivan, Tony R. Merriman, Julia A. Horsfield

**Affiliations:** ^1^Department of Pathology, Dunedin School of Medicine, University of Otago, Dunedin, New Zealand; ^2^Maurice Wilkins Centre for Molecular Biodiscovery, The University of Auckland, Auckland, New Zealand; ^3^Department of Biochemistry, School of Biomedical Sciences, University of Otago, Dunedin, New Zealand; ^4^Liggins Institute, The University of Auckland, Auckland, New Zealand; ^5^Department of Women's and Children's Health, Dunedin School of Medicine, University of Otago, Dunedin, New Zealand

**Keywords:** enhancer, eQTL, gout, HNF4A, lincRNA, *MAF*, non-coding, serum urate

**Addition of an Author**

“Jisha Antony” was not included as an author in the published article. The corrected Author Contributions Statement appears below. The authors apologize for this error and state that this does not change the scientific conclusions of the article in any way. The original article has been updated.

## Author Contributions

ML, JH, JO, and TM designed the study. ML performed the functional annotations, the eQTL analyses, the enhancer assays, the *in situ* hybridization and the siRNA assays. HS isolated and cloned the rs4077450_rs4077451 minor and major allele fragments into the plasmid constructs and was supervised by JM. AD carried out the luciferase assay and was supervised by JA. RT carried out the conditional analysis. WS and TF carried out the CoDeS3D analyses. ML, JH, and TM wrote the manuscript with input from JO and WW.

