# Supplementary material for: Functional Urate-Associated Genetic Variants Influence Expression of lincRNAs LINC01229 and MAFTRR
Source: Front Genet. 2019 Jan 21;9:733. doi: 10.3389/fgene.2018.00733 (PMC6348267; doi:10.3389/fgene.2018.00733)
Supplement: Supplementary file 2 [file Data_Sheet_1.PDF]

Figure S1.

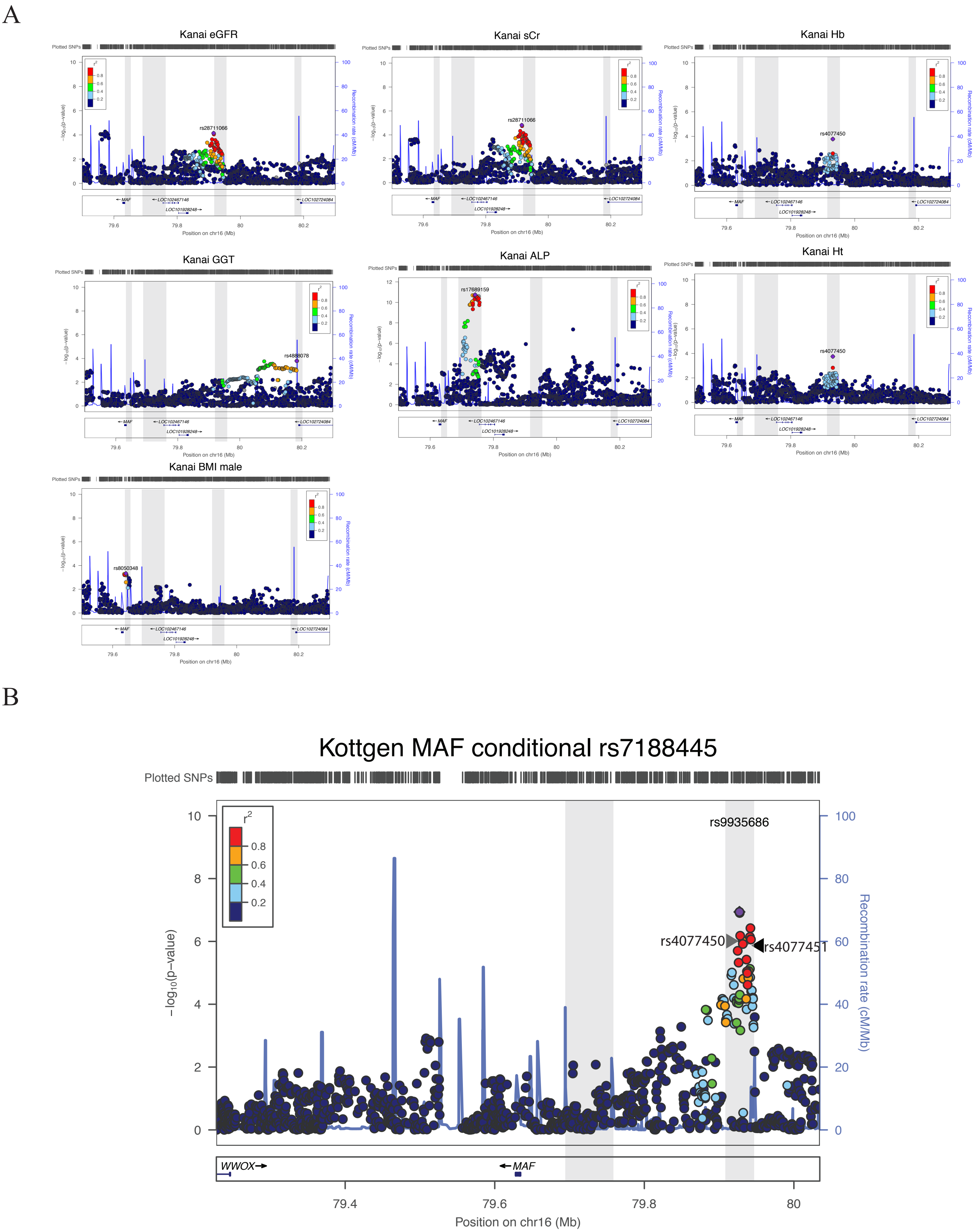

Supplementary Figure 1. **(A)** GWAS from Kanai et al. 2018 at the *MAF* upstream region for kidney-relevant (estimated glomerular filtration rate (eGFR) and serum creatinine (sCr)), liver-relevant (gamma-glutamyl transferase (GGT) and alkaline phophatase levels (ALP)) and blood-relevant (hematocrit (Ht) and hemaglobin (Hb)) traits. **(B)** Regional association plot of a conditional analysis excluding *rs7188445* for serum-urate associated SNPs from Kottgen et al. 2013 upstream of the *MAF* TSS. Each SNP is coloured based on its correlation with the lead SUA2 SNP *rs9935686* in the conditional analysis. Dots represent individual SNPs while their height on the left Y axis indicates significance ($\log_{10}$(p-value)) of association to serum urate level. The blue line indicates recombination rate across the locus. The plots were generated using LocusZoom. The grey boxes indicate SUA1 and SUA2.

Figure S2.

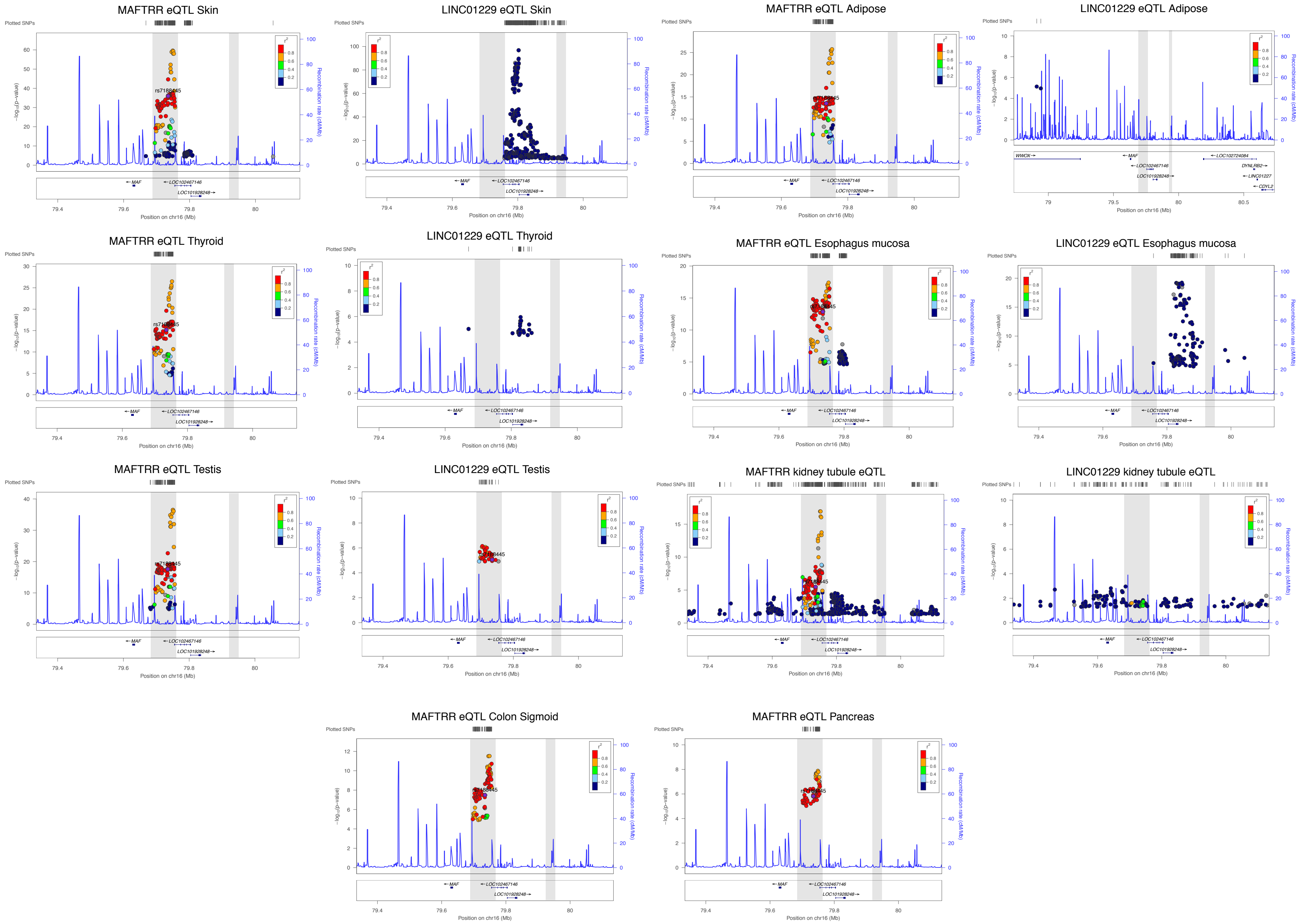

Supplementary Figure 2. GTEx and NepheQTL *MAFTRR* and *LINC01229* eQTL in the genomic region upstream of *MAF*. eQTL plots of SNPs associated with *MAFTRR* and *LINC01229* expression from GTEx (skin sun exposed (lower leg), thyroid, testis, adipose, esophagus mucosa, pancreas and colon (sigmoid)) and NepheQTL (kidney tubule). Each SNP is coloured based on its correlation with the lead SUA1 SNP *rs7188445*. Dots represent individual SNPs while their height on the left Y axis indicates significance ($\log_{10}$(p-value)) of association to *MAFTRR* and *LINC01229* expression. The blue line indicates recombination rate across the locus. The plots were generated using LocusZoom. Grey boxes indicate SUA1 and SUA2.

Figure S3.

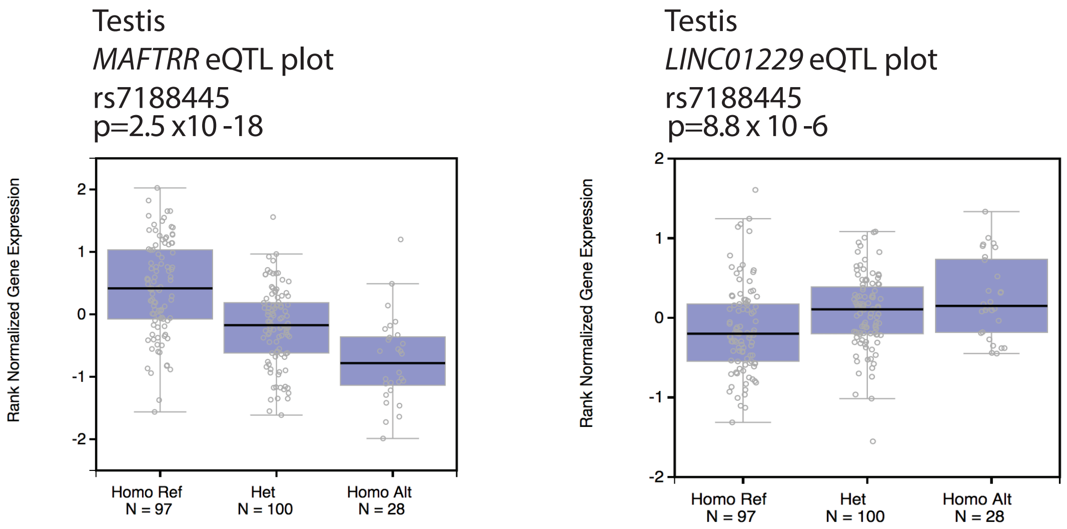

Supplementary Figure 3. eQTL box plots of *rs7188445* for *MAFTRR* and *LINC01229* expression in testis (GTEx). Genotype association of *rs7188445* with *MAFTRR* and *LINC01229* expression. Figure generated from www.gtexportal.org.

Figure S4.

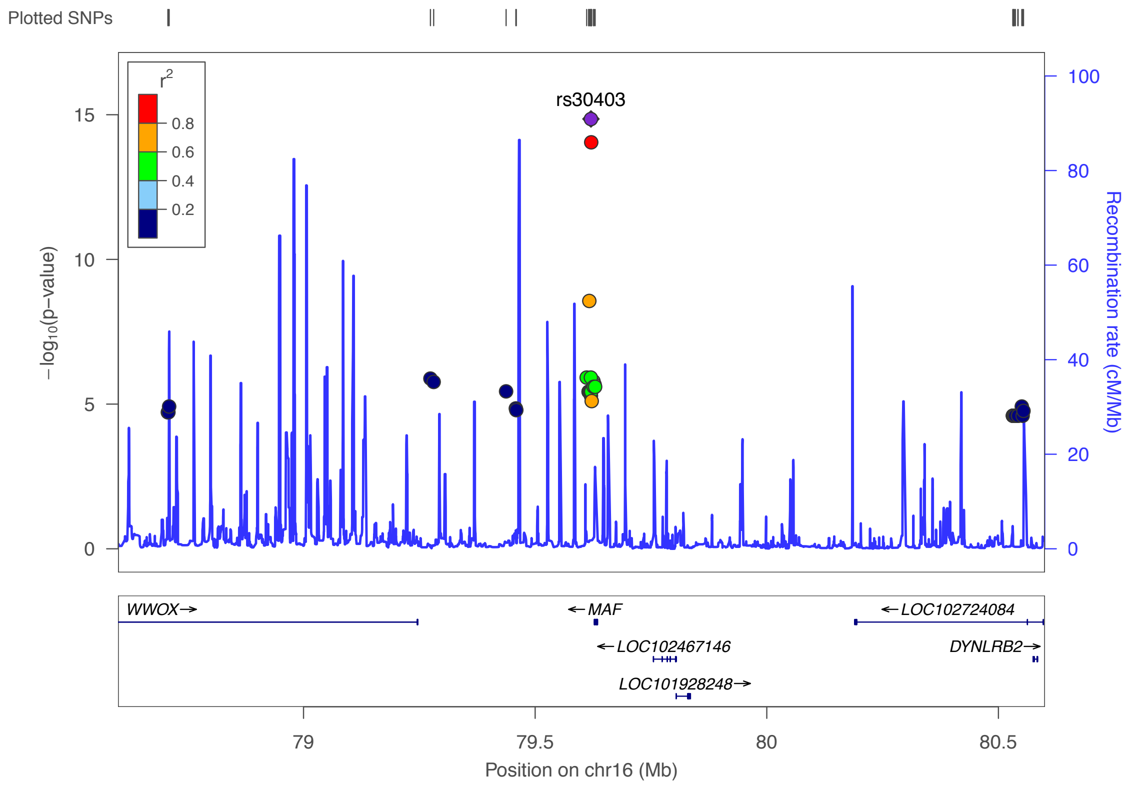

Supplementary Figure 4. GTEx *MAF* eQTL. eQTL plot of SNPs associated with *MAF* expression from GTEx (all tissues). Each SNP is coloured based on its correlation with the lead *MAF* eQTL SNP *rs30403*. Dots represent individual SNPs while their height on the left Y axis indicates significance ($\log_{10}$(p-value)) of association to *MAF* expression. The blue line indicates recombination rate across the locus. The plots were generated using LocusZoom.

Figure S5.

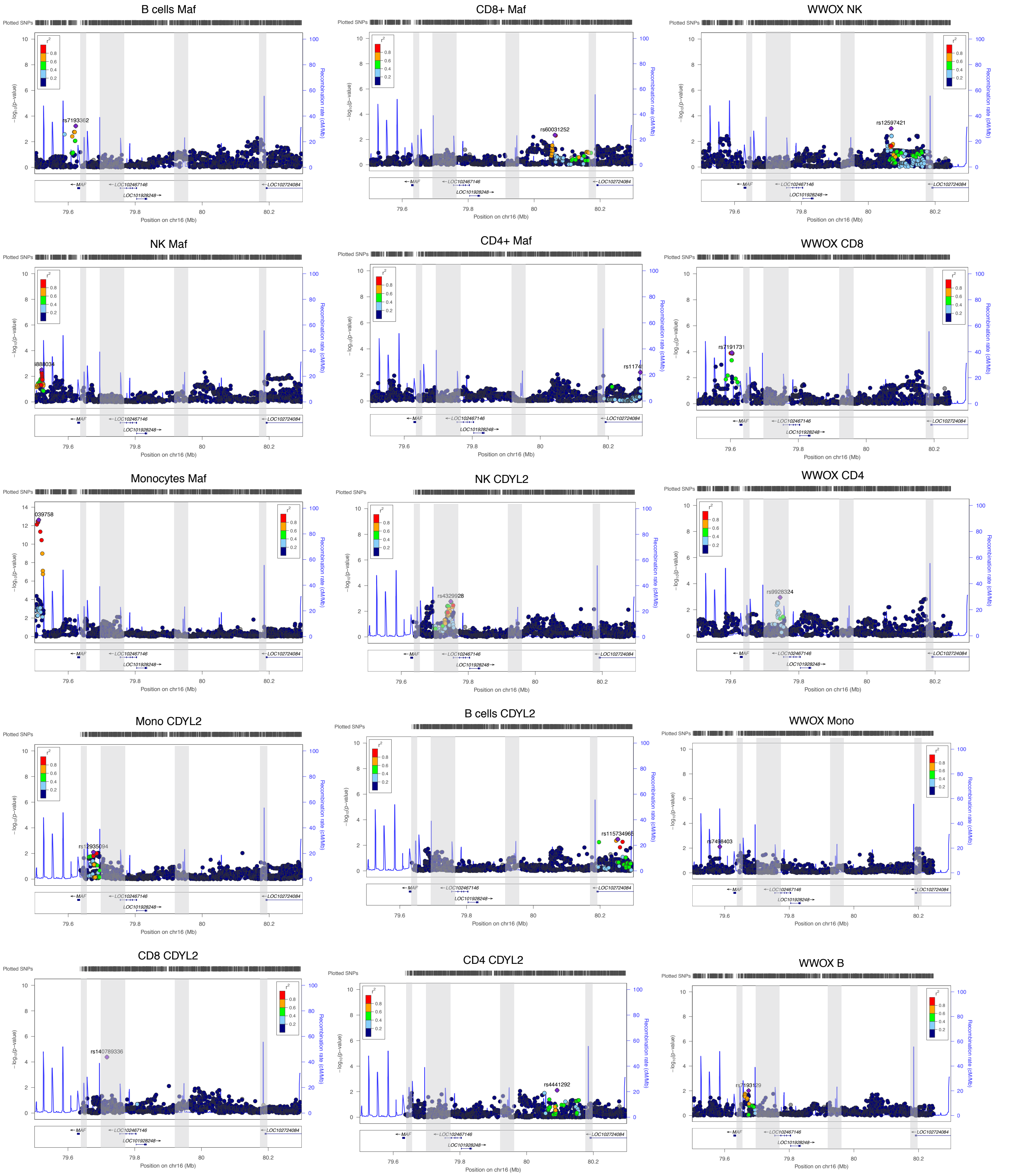

Supplementary Figure 5. eQTL in the genomic region upstream of *MAF* from Kanai et al. 2018. eQTL plots of SNPs associated with gene expression from Kanai et al. 2018 (Monocytes, CD4+, CD8+, Natural Killer cells and B cells). Each SNP is coloured based on its correlation with the lead eQTL SNP. Dots represent individual SNPs while their height on the left Y axis indicates significance ($\log_{10}$(p-value)) of association to gene expression. The blue line indicates recombination rate across the locus. The plots were generated using LocusZoom.

Figure S6.

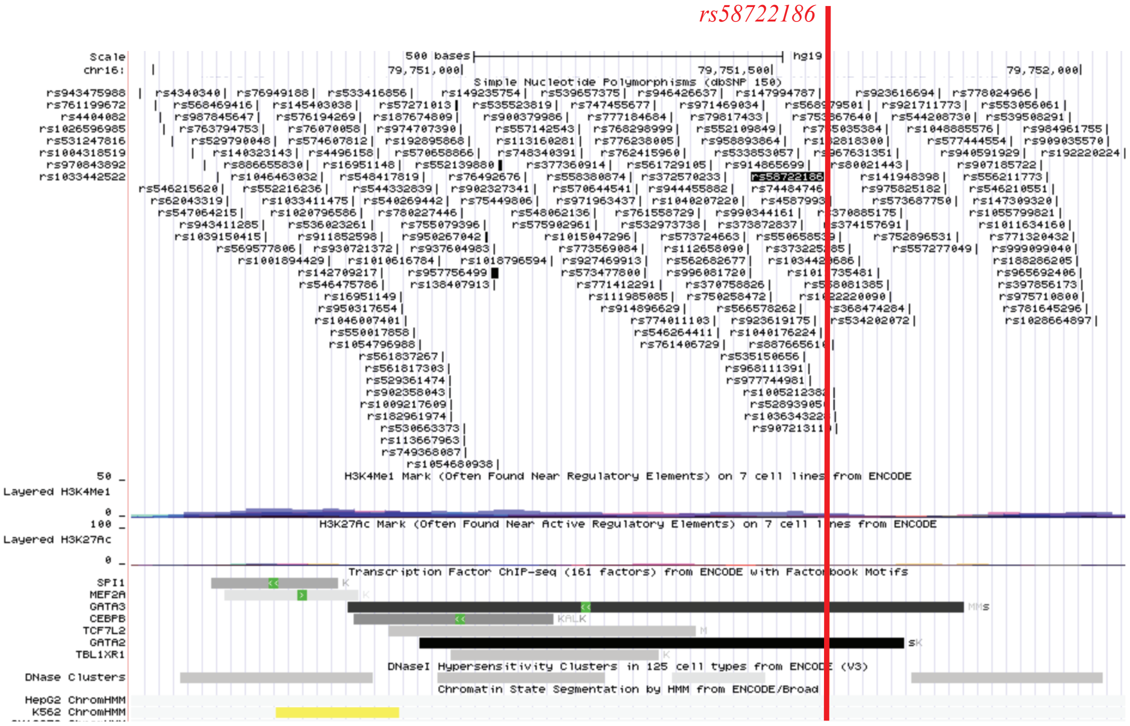

Supplementary Figure 6. The lead PAINTOR SNP in SUA1 marks a weak regulatory region. Screenshot of SUA1 region identified in PAINTOR. SNP region in the UCSC browser (http://genome.ucsc.edu) with dbSNP track, and ENCODE tracks for DHS clusters, layered H3K4me1 and H3K27ac, transcription factor binding sites identified by Factorbook, HepG2 and K562 ChromHMM.

Figure S7.

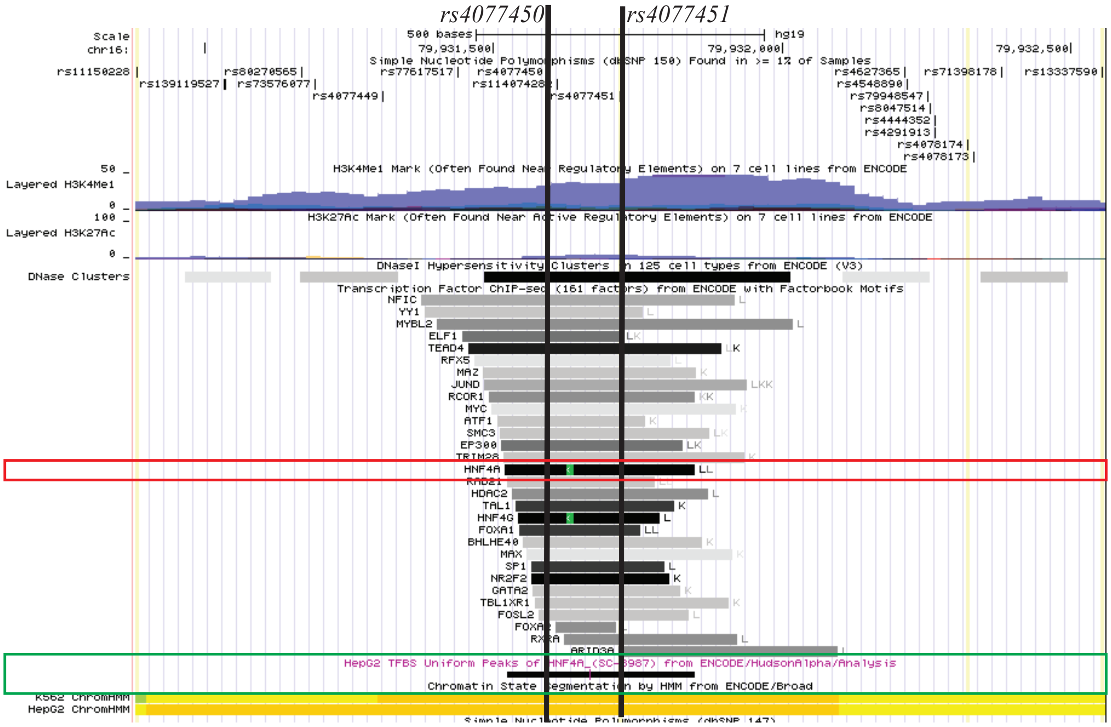

Supplementary Figure 7. The *rs4077450_rs4077451* SNP region in SUA2 marks a putative enhancer element bound by HNF4α. Screenshot of SUA2 region. SNP region in the UCSC browser (http://genome.ucsc.edu) with dbSNP track, and ENCODE tracks for DHS clusters, layered H3K4me1 and H3K27ac, transcription factor binding sites identified by Factorbook (including HNF4α, (red box)), HepG2 and K562 ChromHMM and HepG2 HNF4α ChIP peaks (green box).

Figure S8.

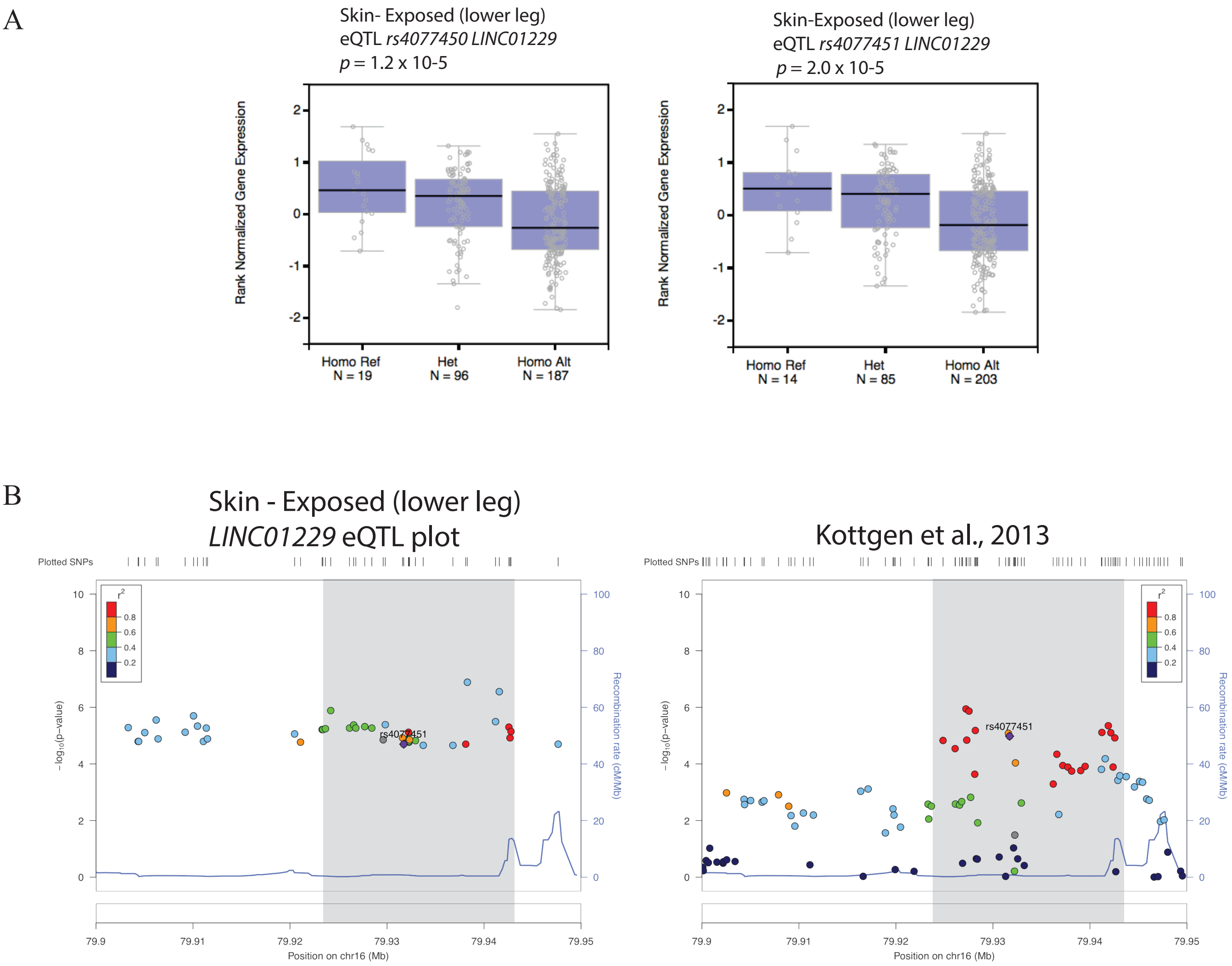

Supplementary Figure 8. eQTL box plots of *rs4077450* and *rs4077451* for *LINC01229* expression in skin sun exposed (lower leg). (A) Genotype association of *rs4077450* and *rs4077451* with *LINC01229* expression in Skin (sun exposed lower leg). *rs4077450* reference allele is T, alternate allele is G. *rs4077451* reference allele is A, alternate allele is T. Figure generated from www.gtexportal.org. **(B)** Association plots of SNPs at SUA2 associated with *LINC01229* expression from GTEx and serum urate (Kottgen et al, 2013). Each SNP is coloured based on its correlation with the SUA2 SNP *rs4077451*. Dots represent individual SNPs while their height on the left Y axis indicates significance ($\log_{10}$(p-value)) of association to *LINC01229* expression. The blue line indicates recombination rate across the locus. The plots were generated using LocusZoom. The grey boxes indicate SUA2.

Figure S9.

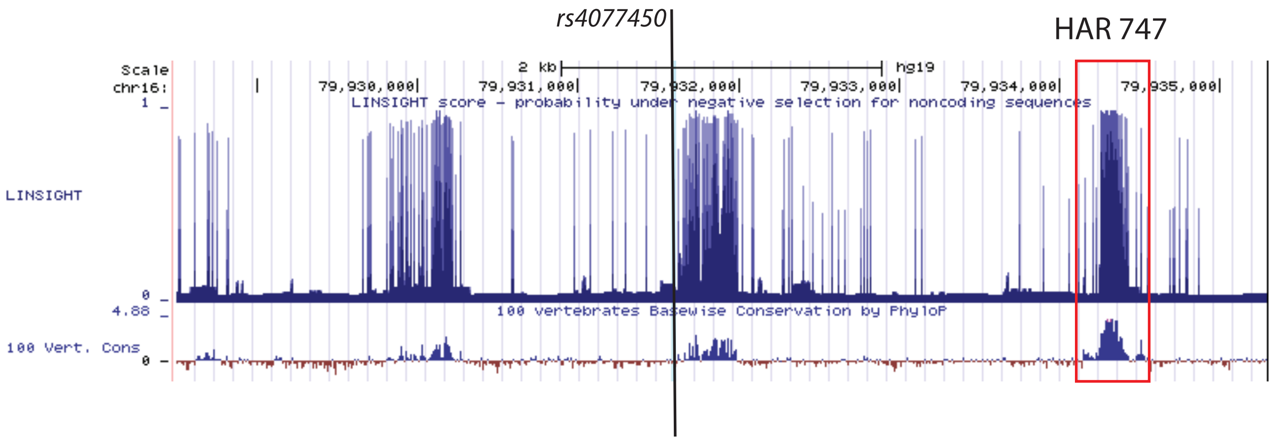

Supplementary Figure 9. SUA2 contains a hyperaccelerated region. A screenshot of the UCSC browser (http://genome.ucsc.edu) with the LINSIGHT track which shows the probability that genomic regions are under negative selection highlights a significant HAR identified in Bird et al. 2007 as HAR 747 ~2 kb upstream of the *rs4077450_rs4077451* SNP region.

Figure S10.

A

B

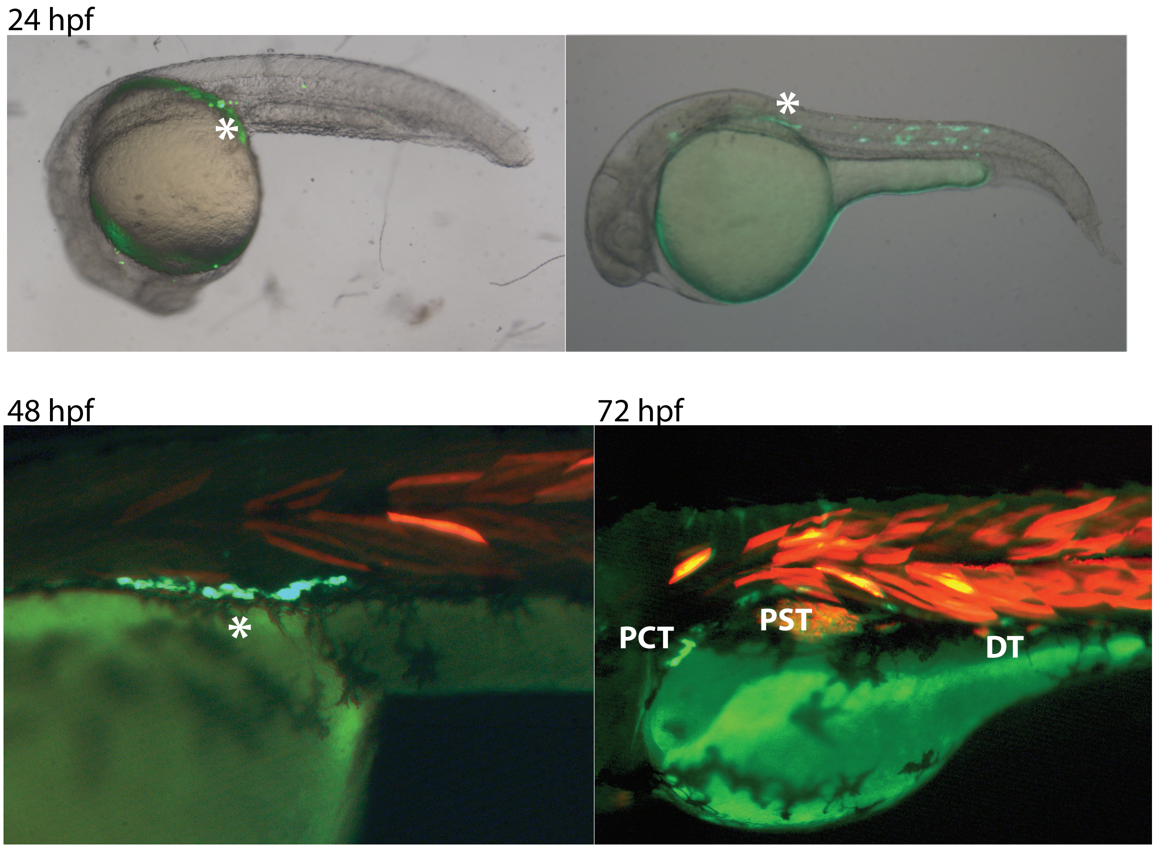

Supplementary Figure 10. Additional images of the enhancer assay. (A) GFP expression is visible in the proximal tubule region (asterisk) in 24 hpf embryos, however the dsRed transgenic marker is not stably expressed at this stage. **(B)** The dsRed transgenic marker is observed as RFP in the somites from 48 hpf onwards. GFP expression is found in the proximal tubule (asterisk) at 48 hpf and the range of GFP expression extends to the proximal convoluted tubule (PCT), proximal straight tubule (PST) and distal tubule (DT) by 72 hpf. Kidney GFP expression does not overlap the RFP signal.

Figure S11.

A

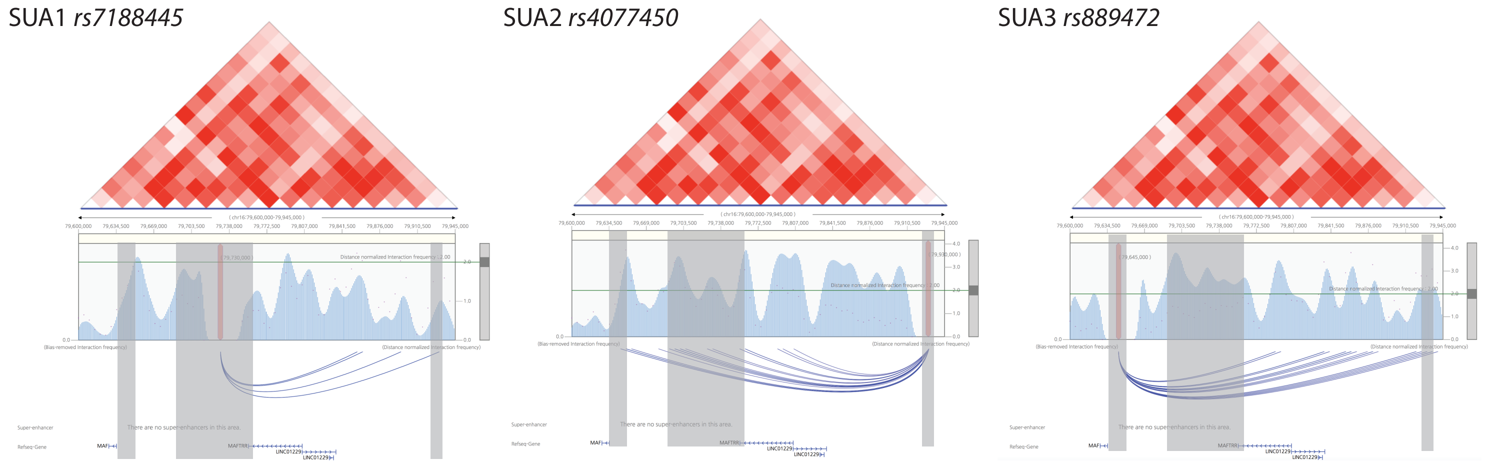

B

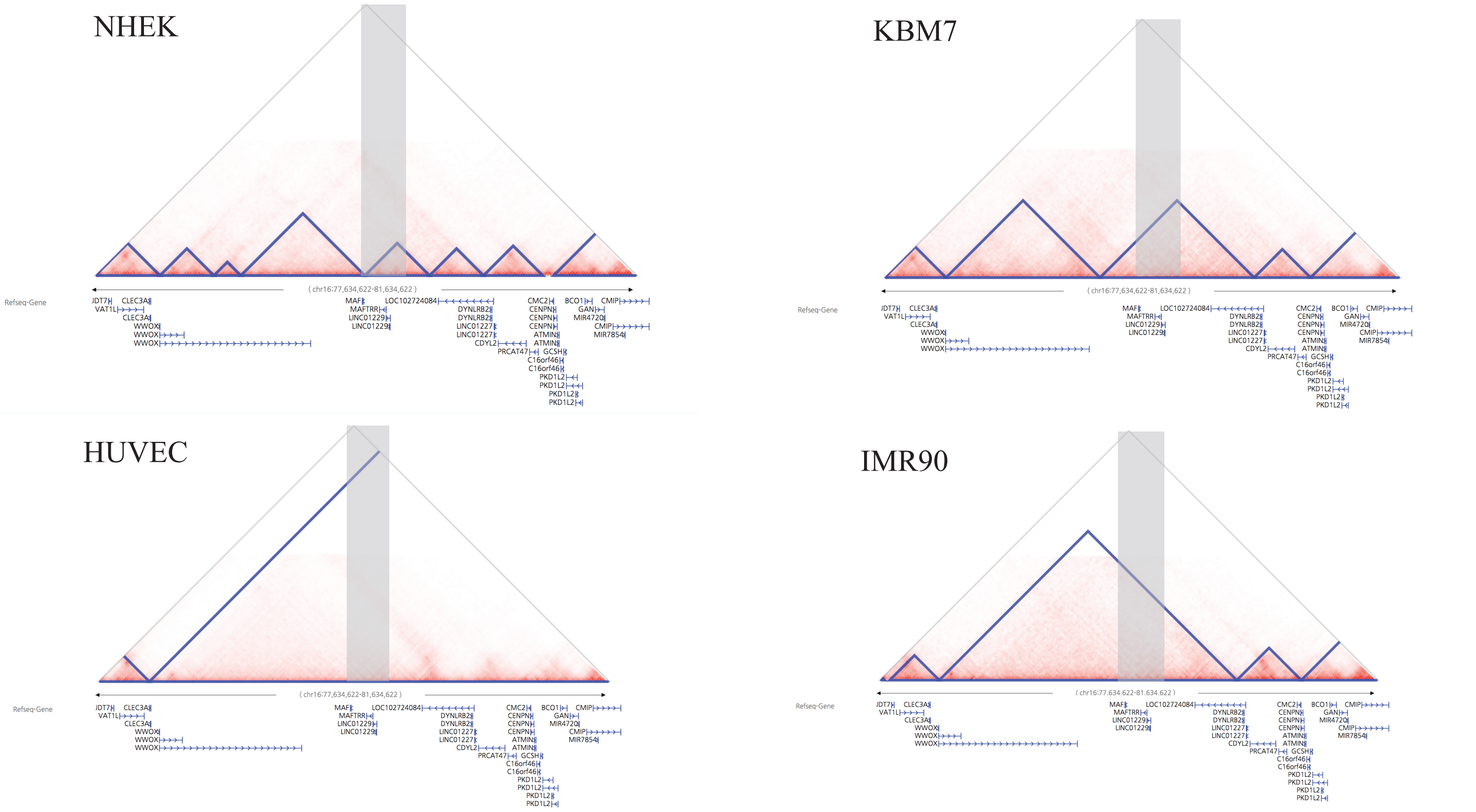

Supplementary Figure 11. HiC reveals interactions and topologically associated domains at the *MAF* locus. (A) HiC data at the lincRNA locus from Caki2 cells showing interactions with SUA1 (*rs7188445*), SUA2 (*rs4077450*) and SUA3 (*rs889472*). **(B)** HiC data for NHEK, KBM7, HUVEC and IMR90 cells indicating the TAD structure at the *MAF* and lincRNA loci. Grey boxes indicate the region encompassing *MAF*, SUA1, the lincRNAs and SUA2.

Figure S12.

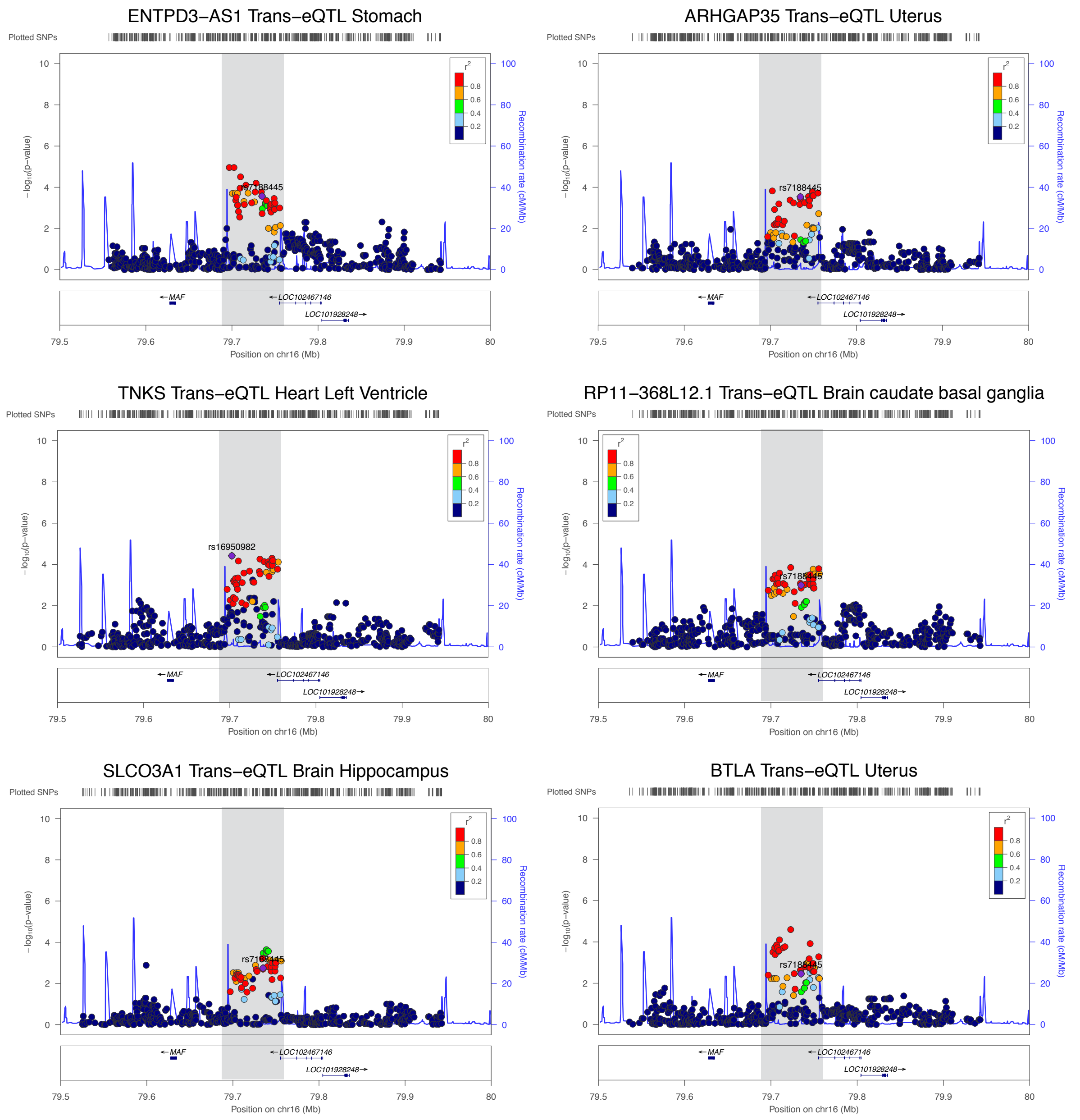

Supplementary Figure 12. Spatial *trans*-eQTL at SUA1. eQTL plots of SNPs associated with *trans*-eQTL expression from GTEx. Each SNP is coloured based on its correlation with the lead urate SUA1 SNP *rs7188445*. Dots represent individual SNPs while their height on the left Y axis indicates significance ($\log_{10}$(p-value)) of association to gene expression. The blue line indicates recombination rate across the locus. The plots were generated using LocusZoom. The grey boxes indicate the location of SUA1.

Figure S13.

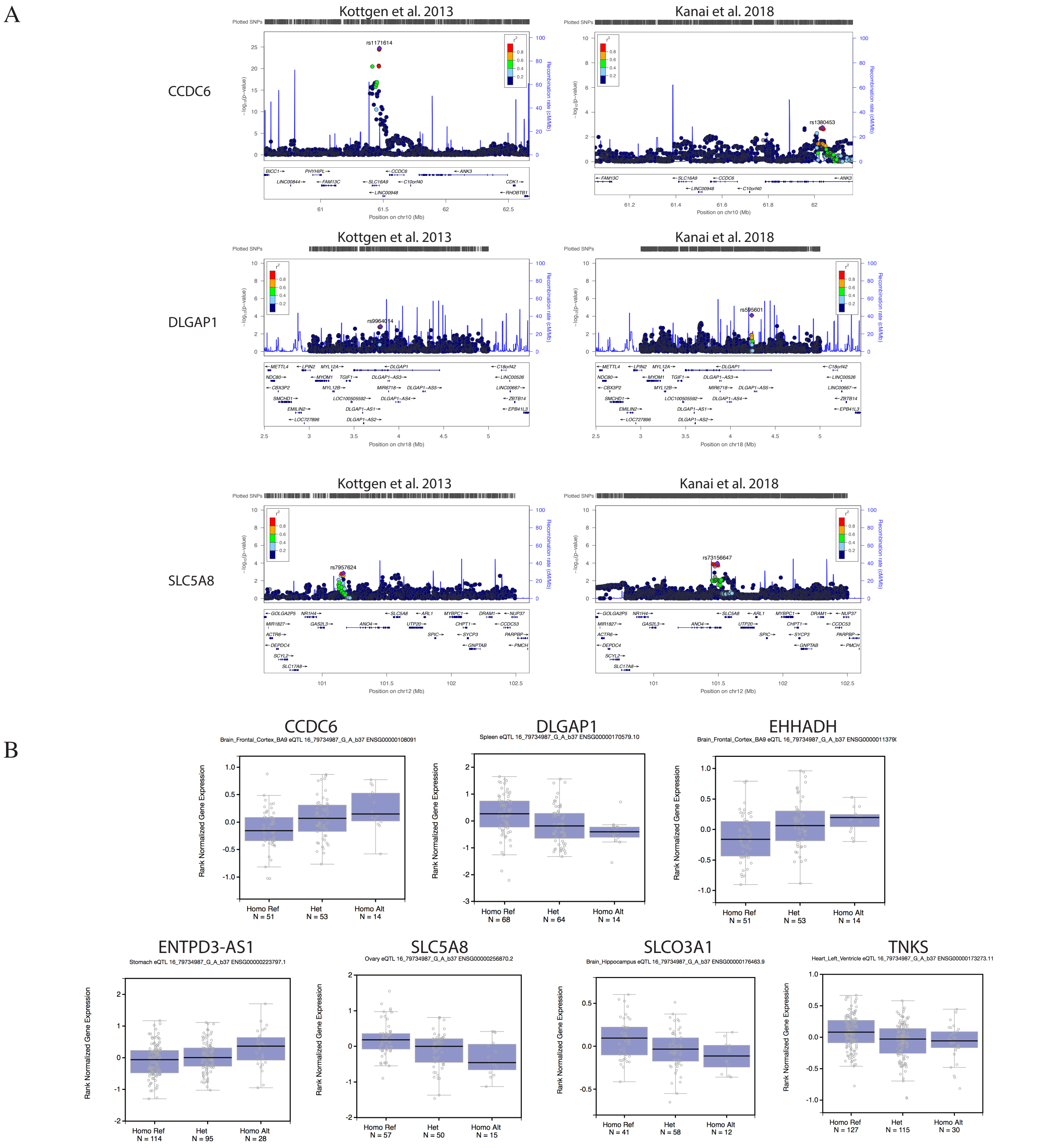

Supplementary Figure 13. *rs7188445* effect on *trans*-eQTL expression and serum urate-associated SNPs in the genomic region of *CCDC6, DLGAP1* and *SLC5A8*. **(A)** Regional association plots of serum-urate associated SNPs from Kottgen et al. 2013 and Kanai et al. 2018 at the *CCDC6, DLGAP1* and *SLC5A8* loci. Each SNP is coloured based on its correlation with the lead SNP. Dots represent individual SNPs while their height on the left Y axis indicates significance ($\log_{10}$(p-value)) of association to serum urate level. The blue line indicates recombination rate across the locus. The plots were generated using LocusZoom. **(B)** eQTL boxplots (GTEx tissue) of *rs7188445 trans*-eQTL indicating effect on expression**.**

Figure S14.

A

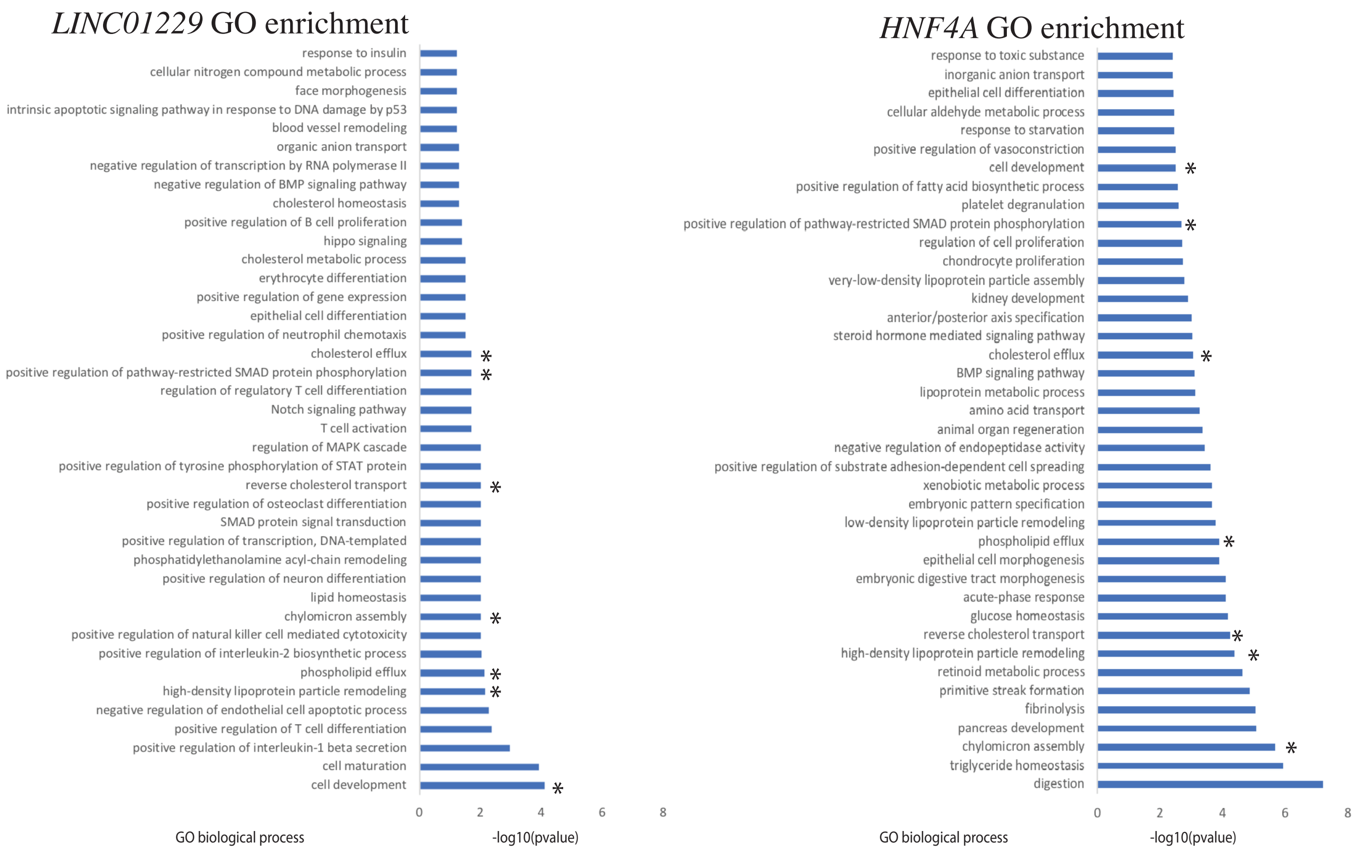

B

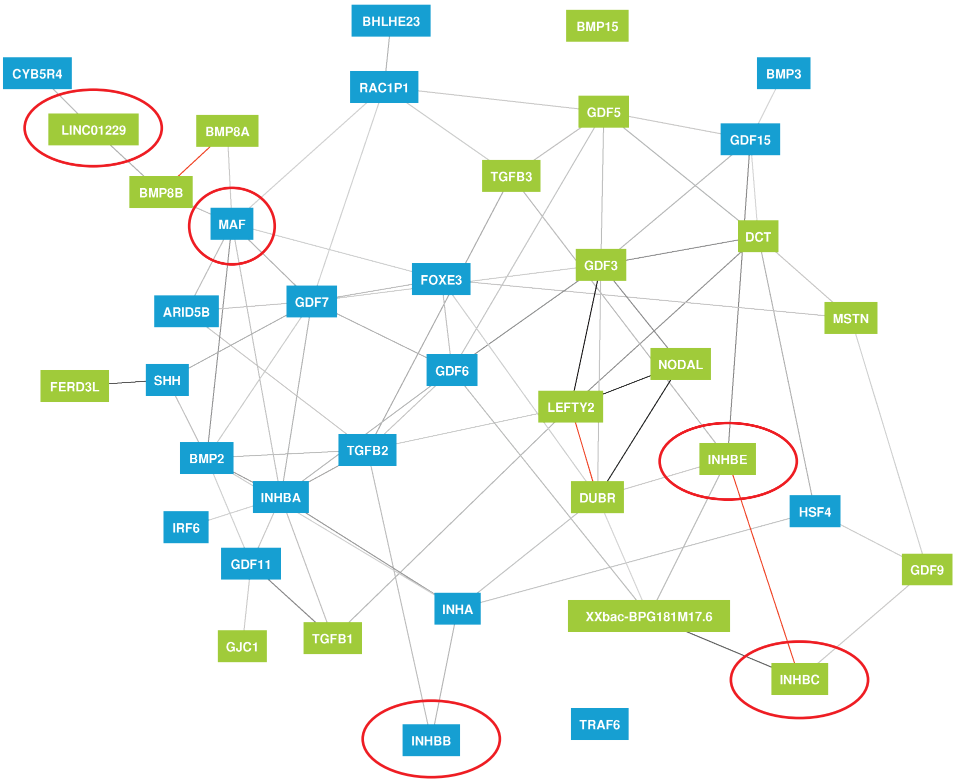

Supplementary Figure 14. GO terms for *LINC01229* and *HNF4A*. (A) Enrichment of GO terms (biological processes) for *LINC01229* and *HNF4A*. based on coexpression data from https://genenetwork.nl/. Asterisk indicate shared GO terms between *HNF4A* and *LINC01229*. (B) Pathway diagram for the top GO biological processes term ‘cell development’ for *LINC01229*. Red circles indicate genes (*LINC01229, MAF, INHBB, INHBC, INHBE*) that have been associated with urate (GWAS).

Figure S15.

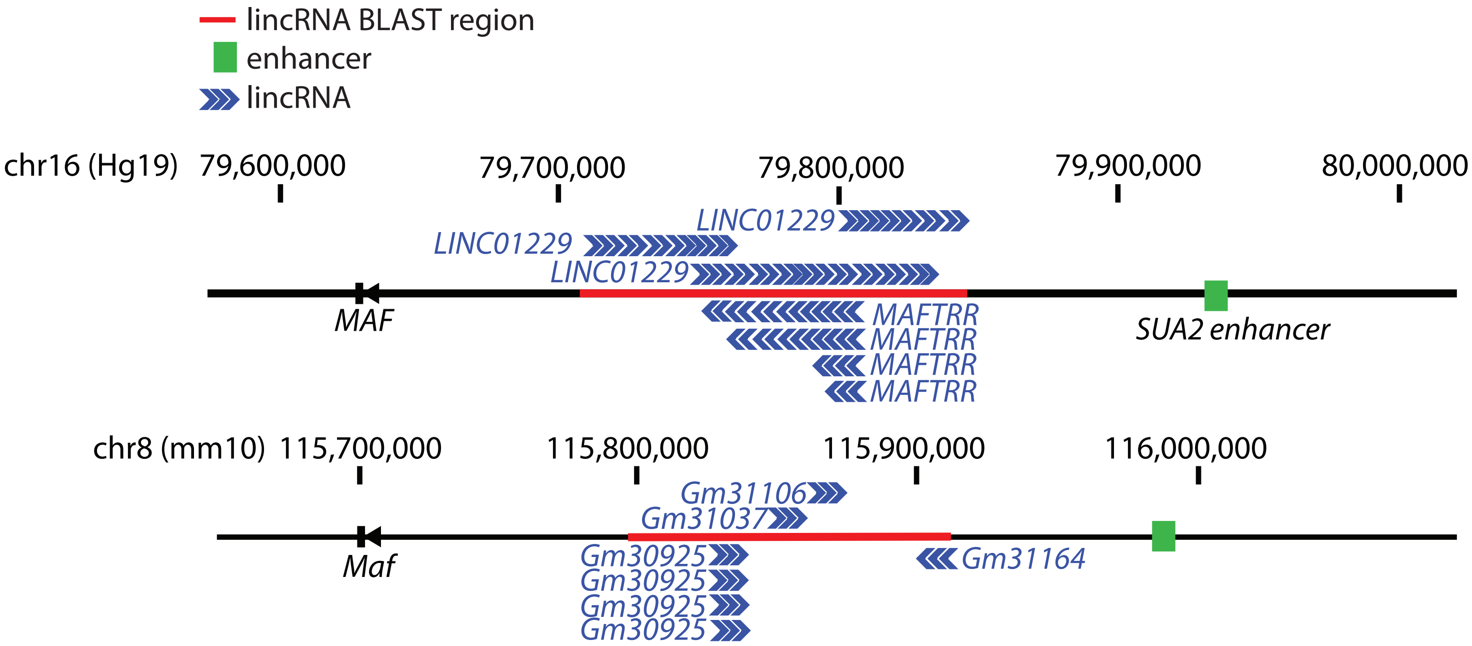

Supplementary Figure 15. Conservation of the *MAF* intergenic genomic regulatory domain. Schematics depict the genomic organisation of the *MAF* locus and the upstream lincRNA genes and regulatory elements on human Chromosome 16 and mouse chromosome 8. The conserved positioning of lincRNAs and the enhancer region upstream of Homo sapiens *MAF* and Mus musculus *maf* together with conservation of sequence indicated by BLAST (red line areas) of the lincRNAs (blue arrows) and enhancer (green box) indicates that the organisation of this genomic regulatory domain is maintained over evolutionary time.
